# COX5B-Mediated Bioenergetic Alteration Regulates Tumor Growth and Migration by Modulating AMPK-UHMK1-ERK Cascade in Hepatoma

**DOI:** 10.3390/cancers12061646

**Published:** 2020-06-22

**Authors:** Yu-De Chu, Wey-Ran Lin, Yang-Hsiang Lin, Wen-Hsin Kuo, Chin-Ju Tseng, Siew-Na Lim, Yen-Lin Huang, Shih-Chiang Huang, Ting-Jung Wu, Kwang-Huei Lin, Chau-Ting Yeh

**Affiliations:** 1 Liver Research Center, Linkou Chang Gung Memorial Hospital, Taoyuan 333, Taiwan; yudechu19871003@gmail.com (Y.-D.C.); victor.wr.lin@gmail.com (W.-R.L.); sam4915@yahoo.com.tw (Y.-H.L.); wunhsin0405@gmail.com (W.-H.K.); wutj5056@gmail.com (T.-J.W.); khlin@mail.cgu.edu.tw (K.-H.L.); 2 Department of Hepatology and Gastroenterology, Linkou Chang Gung Memorial Hospital, Taoyuan 333, Taiwan; 3 Department of Internal Medicine, Chang Gung University College of Medicine, Taoyuan 333, Taiwan; chinchutseng50271@gmail.com (C.-J.T.); siewna.lim@gmail.com (S.-N.L.); 4 Department of Neurology, Linkou Chang Gung Memorial Hospital, Taoyuan 333, Taiwan; 5 Department of Anatomic Pathology, Linkou Chang Gung Memorial Hospital, Taoyuan 333, Taiwan; louisyhuang@gmail.com (Y.-L.H.); ab86401112@adm.cgmh.org.tw (S.-C.H.); 6 Molecular Medicine Research Center, Chang Gung University, Taoyuan 333, Taiwan

**Keywords:** mitochondria, cytochrome *c* oxidase, U2 auxiliary factor homology motif kinase 1, bioenergy, extracellular signal-regulated kinase

## Abstract

The oxidative phosphorylation machinery in mitochondria, which generates the main bioenergy pool in cells, includes four enzyme complexes for electron transport and ATP synthase. Among them, the cytochrome *c* oxidase (COX), which constitutes the fourth complex, has been suggested as the major regulatory site. Recently, abnormalities in COX were linked to tumor progression in several cancers. However, it remains unclear whether COX and its subunits play a role in tumor progression of hepatoma. To search for the key regulatory factor(s) in COX for hepatoma development, in silico analysis using public transcriptomic database followed by validation for postoperative outcome associations using independent in-house patient cohorts was performed. In which, COX5B was highly expressed in hepatoma and associated with unfavorable postoperative prognosis. In addressing the role of COX5B in hepatoma, the loss- and gain-of-function experiments for COX5B were conducted. Consequently, COX5B expression was associated with increased hepatoma cell proliferation, migration and xenograft growth. Downstream effectors searched by cDNA microarray analysis identified UHMK1, an oncogenic protein, which manifested a positively correlated expression level of COX5B. The COX5B-mediated regulatory event on UHMK1 expression was subsequently demonstrated as bioenergetic alteration-dependent activation of AMPK in hepatoma cells. Phosphoproteomic analysis uncovered activation of ERK- and stathmin-mediated pathways downstream of UHMK1. Finally, comprehensive phenotypic assays supported the impacts of COX5B-UHMK1-ERK axis on hepatoma cell growth and migration.

## 1. Introduction

Liver cancer is the seventh most prevalent cancer and the third most frequent cause of cancer-related death in the world [1]. The cause of hepatoma is multifactorial and it often develops against an established background of chronic liver diseases. Generally, chronic hepatitis B and C virus infections, alcoholic hepatitis and non-alcoholic steatohepatitis (NASH), leading to cirrhosis, are all risk factors for hepatoma. To date, surgical removal or non-surgical ablation therapy remains the most efficient curative treatment for hepatoma provided that the diagnosis is made in an early stage. However, it is estimated that only about 20–30% of patients qualify for such treatment [2]. Despite the existing curative treatments, an estimated 50% to 75% of hepatoma patients have already developed cirrhosis [3] and are diagnosed as intermediate- to advanced-stage hepatoma [4]. This situation raises the needs of identifying new druggable targets for treatments.

The intracellular bioenergetic alteration has been implicated in numerous types of cancers owing to its capability to perturb tumor progression. Therefore, the machinery for energy production has emerged as a potential target for anti-cancer or -precancer treatments [5,6,7,8]. The bioenergy is originated from multiple routes, including anaerobic lactate fermentation, aerobic glycolysis, and oxidative phosphorylation (OXPHOS) [9]. According to the theory built-up during the past 80 years as Warburg effect, in cancer cells, shifting bioenergetic metabolism from OXPHOS to aerobic glycolysis is frequently observed especially when dysfunction of respiratory chain occurs in mitochondria [10,11]. However, it has been evident that in addition to the aerobic glycolysis, the OXPHOS also exerts crucial roles in cancer progression [12]. However, whether OXPHOS modulates hepatoma growth has never been clearly delineated, not to mention the possible underlying molecular mechanisms.

OXPHOS provides bioenergy to maintain the progression of intracellular metabolic pathways and thus cell proliferation and survival [13]. It is of particular importance in hepatocytes as liver is one of the major energy-consuming organs. The OXPHOS machinery in mitochondria is composed of five complexes, complex I (NADH: ubiquinone oxidoreductase), complex II (succinate dehydrogenase), complex III (cytochrome *bc*1 complex), complex IV (cytochrome *c* oxidase, COX) and complex V (ATP synthase), and all are localized within the inner mitochondrial membrane [13]. During the process of OXPHOS, protons are pumped by complexes I, III and IV from the mitochondrial matrix to the inter-membrane space, leading to an increase in the membrane potential across the inner mitochondrial membrane. Subsequently, complex V drives the flow of protons back to the matrix, resulting in the generation of ATP from adenosine diphosphate (ADP) [13].

Among these complexes, the COX (complex IV) has been suggested as a major regulatory site of OXPHOS [14,15]. There are at least 13 subunits in this complex, including three mitochondrial DNA (mtDNA) and >10 nuclear DNA encoded subunits. It has been demonstrated that in the macrocomplex, six of these nuclear-encoded subunits could be replaced by isoforms to retain full enzymatic activity, suggesting marked heterogeneity in the composition of this large complex and activity (for a review, see reference [16]). Recently, defects or aberrant expressions of subunits in COX have been associated with clinical prognosis in several types of cancers, including colorectal cancer, glioma, breast and hepatoma [17,18,19,20,21]. However, the actual growth regulatory roles of these subunits have not been clearly investigated. Here, we aim to investigate whether any of the subunits in COX serves as a potential regulator in hepatoma growth.

## 2. Results

### 2.1. COX5B Level is Up-Regulated in Hepatoma and Associated with Unfavorable Postoperative Outcomes

**Figure 1:**
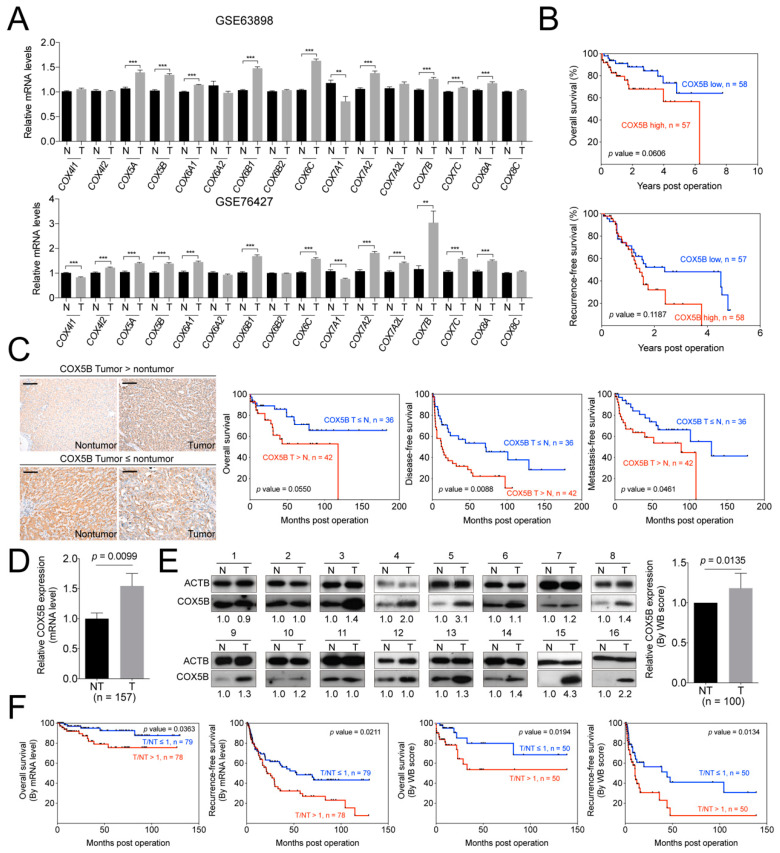
COX5B acts as a potent growth-promoting gene in hepatoma. (**A**) The GSE63898 and GSE76427 datasets were employed to analyze the expression levels of various cytochrome *c* oxidase genes in hepatoma, tumorous versus nontumorous parts. T, tumorous part. N, nontumorous part. ***, *p* < 0.001; **, *p* < 0.01. (**B**) The prognosis of hepatoma patients included in GSE76427 dataset was analyzed by Kaplan-Meyer method with the mean of mRNA levels in the tumorous parts as cutoff. (**C**) The representative IHC sections in patient with higher COX5B intensity in the tumorous tissues (tumor > nontumor, left upper panel) or higher in nontumorous tissues (tumor ≦ nontumor, left lower panel) in hepatoma. The black scale bar represented 100 μm. The Kaplan-Meyer analysis of the prognosis of hepatoma patients stratified according to the intensity of the COX5B IHC staining (right three panels, overall survival, disease-free survival and metastasis-free survival). The (**D**) mRNA and (**E**) protein levels of COX5B in tumorous and nontumorous parts of hepatoma retrieved from our tissue bank. The images in this panel were all acquired by exposing the luminescent signals to X-ray films. The *p* value was derived by paired two-tail student *t*-test. T, tumorous part. N, nontumorous part. (**F**) The Kaplan-Meyer analysis for prognosis of hepatoma patients divided by the mean of the tumorous/nontumorous ratios of COX5B mRNA (left two) and protein (right two) levels.

To interrogate whether any of the nuclear-encoded major subunits in COX complexes served as potential modulators in bioenergetic homeostasis and regulators for hepatocarcinogenesis, the levels of their transcripts were analyzed by employing the published reference datasets, GSE63898 and GSE46727. It was shown in Figure 1A, eight of them, including *COX5A*, *COX5B*, *COX6A1*, *COX6B1*, *COX6C*, *COX7A2*, *COX7B*, *COX7C* and *COX8A*, were consistently elevated, while *COX7A1* was decreased in the tumorous parts, when compared to those in the noncancerous parts. However, only *COX5B* revealed a borderline correlation with the clinical outcome, suggesting a trend of growth promoting function in hepatoma. 

The *p*-value for overall survival (OS) was 0.0606. It was the closest one toward statistical significance among these subunits when transcript levels were used for correlation (Figure 1B and Figure S1). The *p*-value was derived from patients in GSE46727 dataset and grouped by the mean of transcript levels.

To validate these findings and to examine the correlation between COX5B protein levels and clinical outcome, the samples from a cohort of hepatoma patients in our institute were assayed. The IHC staining of COX5B using patient-derived non-tumorous and tumorous parts of tissues were performed and the results were compared according to the staining intensities assessed by two pathologists (Figure 1C). The patients were then divided into two groups by use of the intensities derived from the nontumorous and tumorous parts. As it was shown in Figure 1C, the lower staining intensity of COX5B in tumorous, when compared to nontumorous part, were associated with a better prognosis in OS, recurrence-free survival (RFS) and metastasis-free survival (MFS), albeit a borderline significance in OS. Conversely, the higher intensity in tumorous parts was correlated with a poorer prognosis. The results were consistent with the analysis obtained from the reference datasets, albeit statistically significant.

Encouraged by these finding, validations were further conducted by use of RNA and protein samples from another independent cohort. It was shown in Figure 1D,E, the expression levels of COX5B, either of mRNA or protein, were significantly up-regulated in in the tumorous part of these patients. Moreover, when patients were grouped by high and low tumorous over non-tumorous parts (T/NT) ratios of COX5B for survival curve analyses, it was found that those with T/NT ratio ≥ 1 had a poorer prognostic outcome, while those with ratio < 1, a better outcome. The results were similar in either mRNA or protein level analysis (Figure 1F). These findings strongly suggested that COX5B might be a candidate growth-promoting gene, as its expression was higher in hepatoma and the levels were significantly associated with prognosis.

### 2.2. COX5B Promotes Proliferation and Migration in Hepatoma Cells

**Figure 2:**
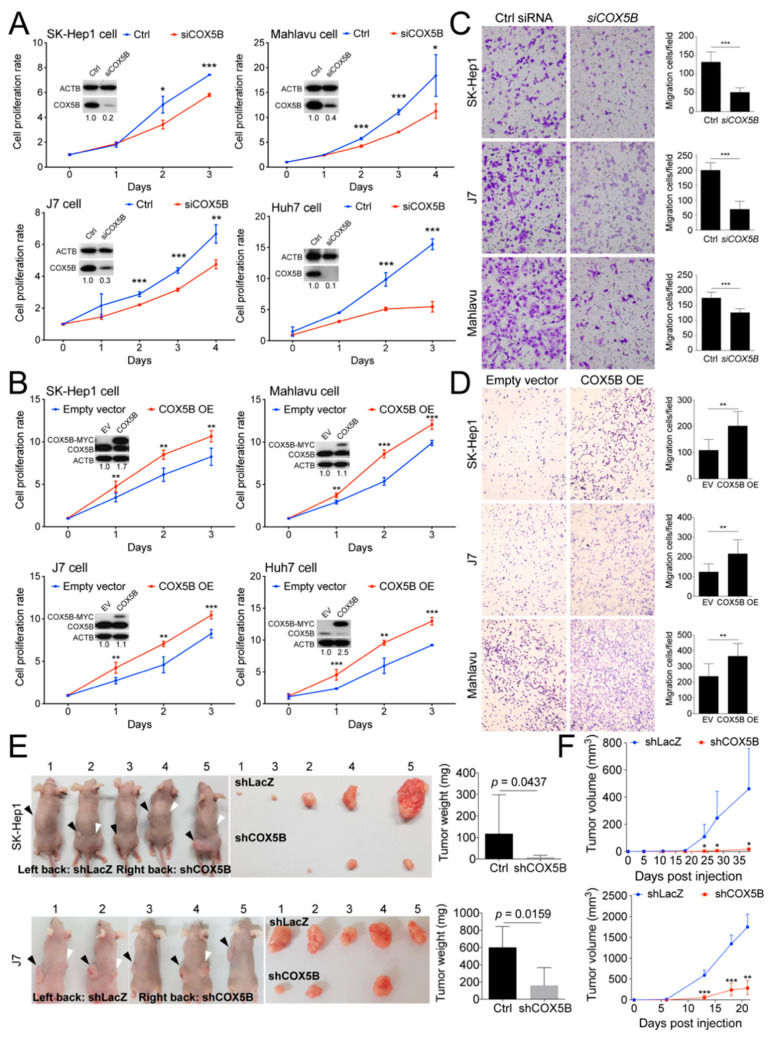
COX5B promotes hepatoma cell growth and migration. Assessment of growth curves in 4 independent hepatoma cell lines was conducted under (**A**) depletion or (**B**) over-expression of COX5B. The western blot images in (**A**,**B**) were all acquired by exposing the luminescent signals to X-ray films. The transwell assays were performed when (**C**) depletion or (**D**) over-expression of COX5B. (**E**) Representative images showing the xenografted tumors 3–6 weeks after implantation of the cells receiving control treatment (shLacZ) or knockdown of COX5B (sh*COX5B*) in SK-Hep1 or J7 cells. The tumors were weighted and statistically compared (right panel). All the in vitro cell-based assays shown in (**A**–**D**) were conducted in duplicates. (**F**) The growth curve of the tumor volume over time was statistically compared. The *p* value was derived from paired two-tail student *t*-test. *, *p* < 0.05; **, *p* < 0.01; ***, *p* < 0.001.

To examine the role of COX5B in tumorigenesis, experimental alterations of its expression levels in hepatoma cells was performed. Three hepatocellular carcinoma cell lines (Mahlavu, J7, and Huh7) and one liver adenocarcinoma cell line (SK-Hep1) were characterized in this study. Firstly, the functional impacts of reducing its level in hepatoma cells was assayed. As was shown in Figure 2A, loss of COX5B expression markedly impeded cell renewal in four hepatoma cell lines. In contrast, overexpressing COX5B markedly elevated cell proliferative rates in these hepatoma cell lines (Figure 2B), suggesting that COX5B expression enhanced cell proliferation in hepatoma cells. To test whether expression of COX5B affected cell migration and invasion in hepatoma, the transwell assay was conducted. As shown in Figure 2C,D, it was discovered that knock-down of COX5B significantly repressed cell migration, while increasing its expression enhanced cell migration in hepatoma cell lines. Consistently, the reduced level of Snail, the classical marker for cell migration, also supported that silencing COX5B in hepatoma restrained cell migration (Figure S2). Additionally, the results of xenograft experiments were in agreement with the notion that reduced COX5B expression impeded tumorigenesis in hepatoma (Figure 2E,F).

### 2.3. Loss of COX5B Represses Cell Proliferation Partly through Induction of Cell Senescence

**Figure 3:**
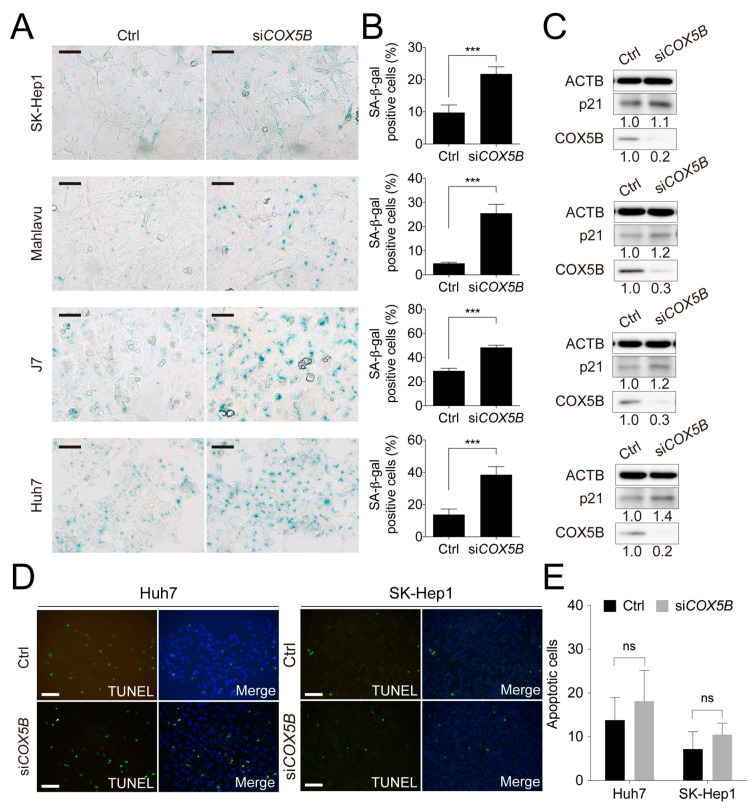
Knockdown of COX5B suppresses cell proliferation through induction of cell senescence but not programmed cell death. This figure was used to supplement Figure 2 in the main text. (**A**) The beta-galactosidase staining assay to examine the status of cell senescence. The black Scale bar represented 50 μm. The quantitative results were shown in (**B**). ***, *p* < 0.001. (**C**) The western blot analysis of cell senescence marker p21 levels in HCC cells with or without depletion of COX5B. The western blot images in this panel were all acquired by using chemStudio PULS imaging system. (**D**) The TUNEL assay to determine the status of cell apoptosis. The white scale bar represented 50 μm. The quantitative results were shown in (**E**). All the in vitro cell-based assays shown in this figure were conducted in duplicates.

Previously, a report regarding the role of COX5B in breast cancer demonstrated that loss of COX5B function led to cell senescence [22]. To examine whether the status of cell senescence was influenced under lowering COX5B expression in hepatoma, the beta-galactosidase staining assay was conducted. Indeed, as was shown in Figure 3A,B, decreasing COX5B level greatly enhanced cells toward senescence. The increased p21 protein level, a marker for senescent cells, also supported this notion (Figure 3C). Conversely, no significant difference in the apoptotic status was found under the same situation in hepatoma cells (Figure 3D,E), indicating that reducing COX5B-mediated decline of proliferative potential was not a result of enhanced cell apoptosis but a consequence of increased cell senescence.

### 2.4. COX5B Modulates an Oncogene UHMK1 and a Potent Tumor Suppressor ULK1 Expressions in Hepatoma

**Figure 4:**
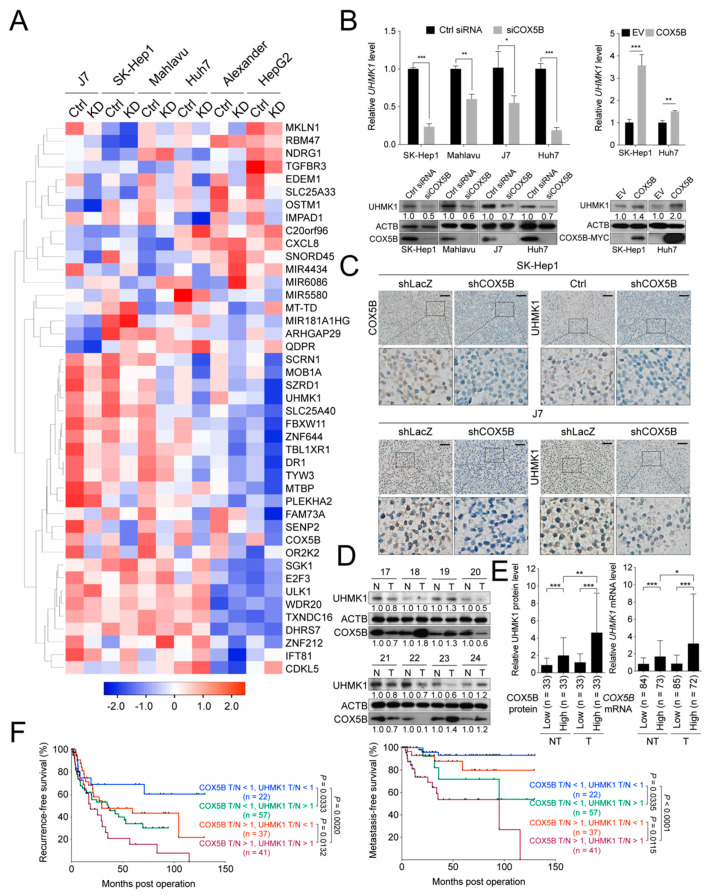
COX5B modulates UHMK1 expression in hepatoma. (**A**) The heat map representation of the relative gene expression levels in hepatoma cells receiving control treatment (Ctrl) and knockdown of COX5B (KD). The average linkage method was used for hierarchical clustering. The distances between rows and columns were computed by Pearson method. (**B**) RT-qPCR and western blot were performed for validation of UHMK1 expression changes under depletion of COX5B. All the in vitro cell-based assays shown in (**B**) were conducted in duplicates. (**C**) IHC staining of paraffin-embedded tissues derived from Figure 2E. The expression relationships between COX5B and UHMK1 in (**D**) protein and (**E**) mRNA levels in samples derived from hepatoma patients. The *p* value was derived from paired two-tail student *t*-test. *, *p* < 0.05; **, *p* < 0.01; ***, *p* < 0.001. The western blot images in (**B**,**D**) were all acquired by exposing the luminescent signals to X-ray films. (**F**) The Kaplan-Meyer analysis of recurrence-free and metastasis-free survival in hepatoma patients stratified according to the T/N ratio of *COX5B* and *UHMK1* mRNA levels.

Subsequently, to assess the downstream effector that is responsible for COX5B-mediated regulation of tumorigenesis, the transcriptomic cDNA microarray was performed. There were 6 distinct hepatoma cell lines, including J7, SK-Hep1, Mahlavu, Huh7, Alexander and HepG2, utilized in this experiment, comparing between cells with and without COX5B knockdown. Among the transcripts with significant change, U2AF homology motif kinase1 (UHMK1), an oncogene, was down-regulated and Unc-51 like autophagy activating kinase 1 (ULK1), a potential tumor suppressive gene, was up-regulated in the most significant and consistent manner in all hepatoma cell lines (Figure 4A). 

The mRNA and protein levels of UHMK1 and ULK1 were then examined for validation by using samples from COX5B downregulated or overexpressed hepatoma cells. As was shown in Figure 4B and Figure S3A,B, the levels of UHMK1, mRNA and protein, were positively, while those of ULK1 were negatively correlated with COX5B expression, suggesting that UHMK1 and ULK1 might be the downstream effectors of COX5B in modulating cell growth and migration. Moreover, a similar tendency was also observed when examining their relationships by using tissues derived from xenograft experiments (Figure 4C and Figure S3C).

Next, to determine whether the relationship between COX5B and UHMK1 could also be observed in samples from patients with hepatoma, the western blot and qRT-PCR were conducted. As was shown in Figure 4D,E, the COX5B expression was significantly and positively correlated with the levels of UHMK1 in hepatoma tissues. Moreover, further stratification of patients into four groups by COX5B and UHMK1 levels revealed that those with both higher COX5B and UHMK1 expression manifested the poorest prognosis (recurrence-free survival and metastasis-free survival), while those with lower expression of both displayed the best prognosis. Moderate outcomes were observed in those with either higher COX5B but lower UHMK1 or lower COX5B but higher UHMK1 expression (Figure 4F), suggesting the COX5B-UHMK1 axis might be crucial for hepatoma progression. Nevertheless, no significant difference was found when comparing the overall survival rates in these groups (Figure S4).

### 2.5. COX5B-Mediated Regulation of UHMK1 and ULK1 Expression May Be AMPK Activation-Dependent

**Figure 5:**
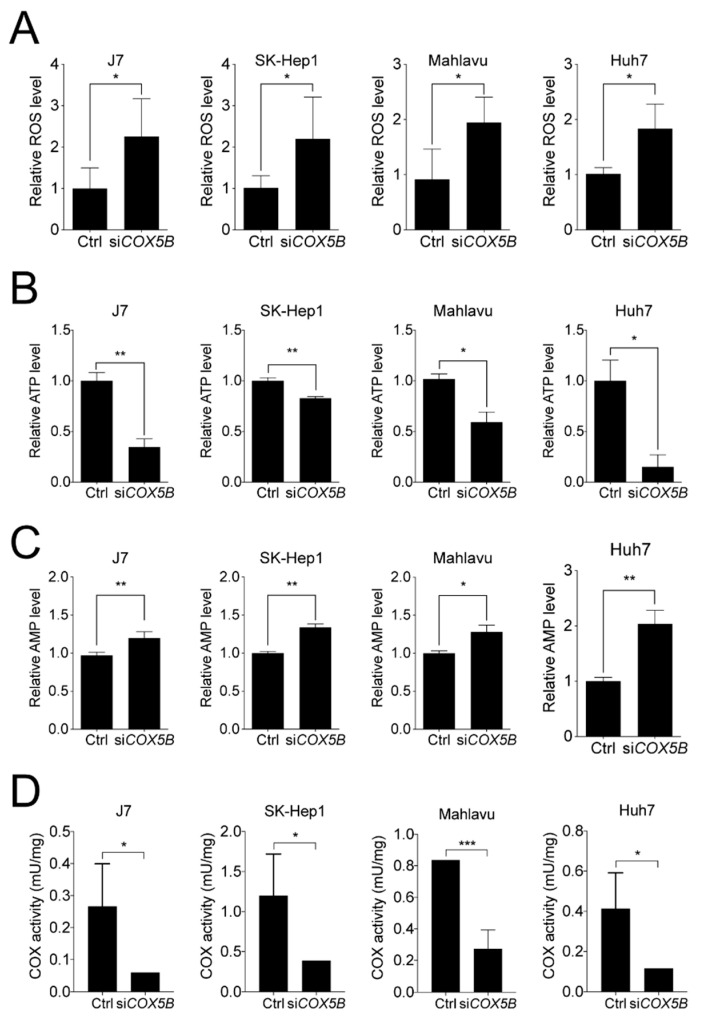
Loss-of-function in COX5B leads to dysfunction of mitochondria and COX. The function of mitochondria was assessed by measuring (**A**) ROS, (**B**) ATP and (**C**) AMP levels with or without COX5B depletion in hepatoma cells. (**D**) The COX activity to oxidize cytochrome c was measured using mitochondria solution isolated from indicated hepatoma cells with or without COX5B silencing. The *p* value was derived from paired student *t*-test (two tails). *, *p* < 0.05; **, *p* < 0.01; ***, *p* < 0.001.

Since COX5B is a mitochondrial protein, it was mandatory to examine whether abnormal expression of COX5B led to aberrant accumulation of reactive oxygen species (ROS) and unbalanced production of bioenergy, the main types of stress originated from dysfunction of mitochondria. To investigate this issue, the levels of ATP and ROS were assessed with or without COX5B knockdown. As was demonstrated in Figure 5A,B, the levels of ROS were markedly elevated while the levels of ATP were decreased in hepatoma cells when knocking down COX5B. Theoretically, the decreased ATP levels in COX5B-depleted cells would lead to an elevated amount of adenosine monophosphate (AMP) in cells. To confirm this, the AMP levels were assessed in cells with or without COX5B depletion. Indeed, it was found that AMP levels were significantly elevated in those with lowered COX5B expression (Figure 5C). Additionally, to examine whether loss of COX5B expression indeed impacted COX function, the activity of the complex IV in OXPHOS was measured. As was shown in Figure 5D, the ability of COX to oxidize cytochrome c was significantly decreased when hepatoma cells were under COX5B knockdown. These findings indicated that the cells were suffering from a stressed condition upon COX5B silencing, where it was unable to cause cell apoptosis but it led to cell senescence (Figure 3).

According to the Warburg effect, dysfunction of OXPHOS may lead to changes in the sources of bioenergetic production from OXPHOS to aerobic glycolysis. Examination of the capabilities of bioenergy production from either the respiratory chain or glycolysis, with or without COX5B silencing, was conducted by using the seahorse assay. As was shown in Figure S5A, reduced basal and maximal respirations and proton leak were consistently observed in cells with silenced COX5B expression, indicating a significant reduction in the function of respiratory chain. However, only knockdown of COX5B in Huh7 cells exhibited an elevated glycolysis capacity, while no difference was observed in the other cell lines (Figure S5B), suggesting a minor or limited role of COX5B activity lost in promoting shift of bioenergy metabolism from OXPHOS to aerobic glycolysis.

**Figure 6:**
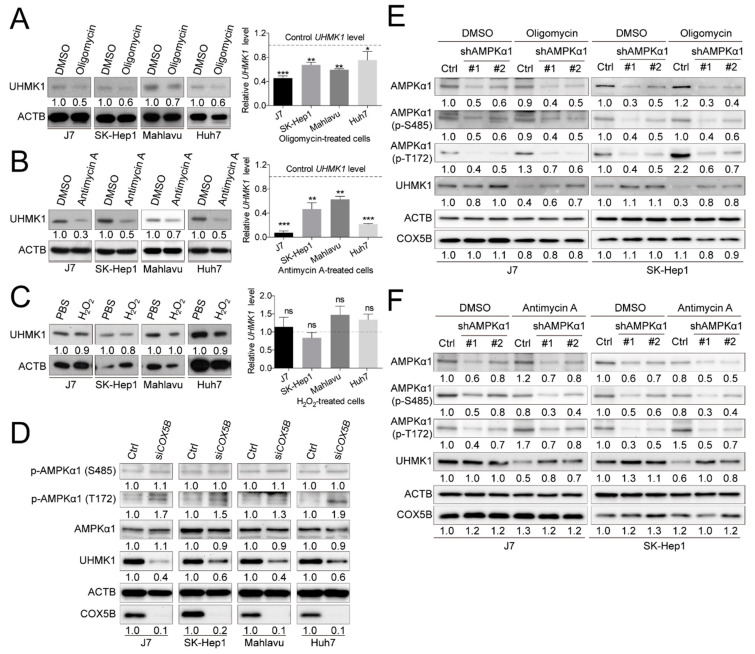
COX5B-mediated bioenergetic alteration modulating UHMK1 expression is AMPK activation-dependent. Western blot and RT-qPCR analysis of cell lysates derived from cells treated with (**A**) oligomycin, (**B**) antimycin A and (**C**) H_2_O_2_. Oligomycin and antimycin A were used for blocking bioenergy production. H_2_O_2_ was used for accumulating ROS. The western blot images in this panel were acquired by exposing the luminescent signals to X-ray films, except for those of the Antimycin-treated Mahlavu cell, which was obtained by using chemStudio PLUS imaging system. The *p* value was derived from paired two-tail student *t*-test. *, *p* < 0.05; **, *p* < 0.01; ***, *p* < 0.001; ns, not significant. (**D**) The relationship between depletion of COX5B and activation of AMPK was assessed by western blot. AMPK T172 phosphorylation indicated activation while S485 phosphorylation was considered suppression of enzyme activities. The combined analysis of the relationship between bioenergetic alteration, induction by (**E**) oligomycin and (**F**) antimycin A, and activation of AMPK in modulation of UHMK1 expression. The western blot images in (**D–F**) were all acquired by using chemStudio PULS imaging system. All the in vitro cell-based assays shown in this figure were conducted in duplicates.

Subsequently, to address the question of whether reduction of UHMK1 and up-regulation of ULK1 levels under COX5B depletion were a consequence of aberrantly homeostatic control of bioenergy or a result of increased ROS (or both), the cells were treated with ATP production-inhibitors or ROS-induced chemical for subsequent analyses. It was discovered that restraining generation of ATP by antimycin A and oligomycin, but not inducing accumulation of ROS by H_2_O_2_, resulted in repressed UHMK1 and enhanced ULK1 expression in hepatoma cells (Figure 6A–C and Figure S6), suggesting that COX5B-mediated UHMK1 and ULK1 expression regulation was a result of bioenergetic alteration.

Increased AMP/ATP ratio has been well-recognized as a prerequisite for activation of AMP-activated protein kinase (AMPK) [23]. To examine whether COX5B-depletion induced activation of AMPK, western blot analysis was conducted to examine the phosphorylation status of well-defined amino acid residues in AMPKα1, a major subunit in AMPK. As was shown in Figure 6D, when depleting COX5B, phosphorylation at Thr172 in AMPKα1, which resulted in activation of AMPK, was markedly increased, whereas only little or no phosphorylation change was found on Ser485, which led to inhibition of AMPK activity [24]. A similar tendency was also observed in IHC staining results by using tissues from xenograft (Figure S7). To further explore the issue of whether activation of AMPK was required for COX5B-mediated regulation of UHMK1 and ULK1 expression, western blot analysis was performed by using samples from cells treated with bioenergy production inhibitors, oligomycin or antimycin A, with or without AMPKα1 depletion. Interestingly, as was shown in Figure 6E,F and Figure S8, the oligomycin or antimycin A induced reduction of UHMK1 and increase of ULK1 levels were partially relieved by simultaneous knockdown of AMPKα1, suggesting that the COX5B-mediated regulation of UHMK1 and ULK1 expression was AMPK activation-dependent.

### 2.6. COX5B-UHMK1 Axis Modulates Cell Proliferation and Migration in Hepatoma

**Figure 7:**
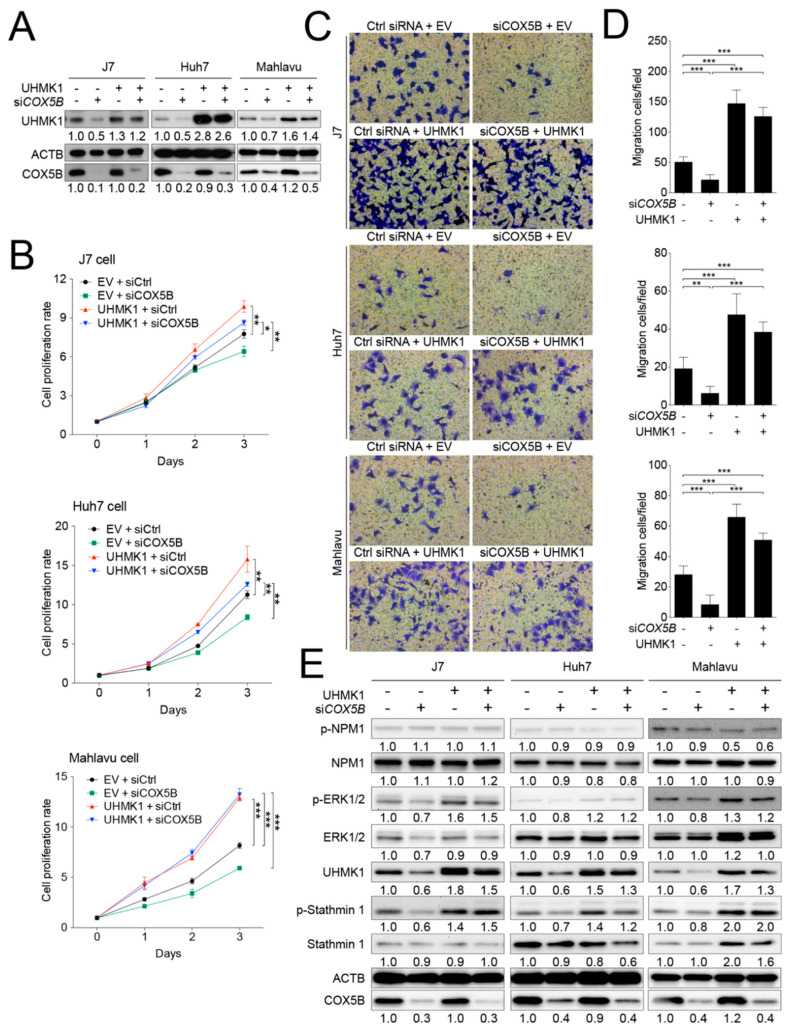
COX5B-UHMK1 axis is crucial for regulating cell proliferation and migration in hepatoma cells. (**A**) The western blot analysis of UHMK1 in 3 independent hepatoma cell lines receiving the indicated treatments. The western blot images in this panel were acquired by exposing the luminescent signals to X-ray films, except for those of Mahlavu cell, which were obtained by using chemStudio PLUS imaging system. (**B**) The growth curves of hepatoma cells receiving the indicated treatments. (**C**) The transwell-based assays to evaluate cell migration capability under the indicated treatments. The quantitative results were shown in (**D**). The *p* value was derived from paired two-tail student *t*-test. *, *p* < 0.05; **, *p* < 0.01; ***, *p* < 0.001. (**E**) The western blot analysis of the potentially phosphorylated candidates downstream of UHMK1 in hepatoma cells. The western blot images in (**E**) were all acquired by using chemStudio PULS imaging system. All the in vitro cell-based assays shown in this figure were conducted in duplicates.

Next, to examine whether UHMK1 indeed served as a downstream effector of COX5B in modulating hepatoma progression, the rescue experiment was conducted by overexpressing UHMK1 in cells with or without siRNA knockdown of *COX5B* (Figure 7A). The cell proliferation curves were depicted, which revealed that overexpressing UHMK1 relieved the proliferative capability of cells restrained by COX5B knockdown (Figure 7B). Similarly, the migratory ability was also rescued by elevating UHMK1 expression in hepatoma cells under COX5B depletion (Figure 6C,D). These results strongly suggested that UHMK1 indeed functioned downstream of COX5B to modulate cell renewal and migration in hepatoma.

### 2.7. Elevation of UHMK1 Promotes Phosphorylation of ERK1/2 and Stathmin1 in Hepatoma Cells

As UHMK1 is a kinase, it is possible that UHMK1 impacted hepatoma cells proliferation and migration via modulating hepatoma growth-related protein(s) or pathway(s). As such, the J7 cells-derived protein lysates (as used in Figure 7A experiments) were subjected to phospho-peptides enrichment analysis to search for potent UHMK1 downstream target(s). As listed in Table 1, a panel of potential downstream phospho-proteins were identified by comparing the ratio of phospho-peptides enriched between groups. Among them, phosphorylation occurred at specific sites in ERK2, Stathmin1 and NPM1 were most significantly changed between groups. Validations were conducted by using 3 different hepatoma cell lines via western blot analysis. As was shown in Figure 7E, the levels of phosphorylated ERK2 and Stathmin1 were markedly reduced when depleting COX5B, whereas the levels were recovered or elevated under simultaneous overexpression of UHMK1. However, such effect was not observed in the levels of phosphorylated NPM1. These results implied that activation of ERK2 and Stathmin1 through UHMK1-dependent phosphorylation might be required for downstream events caused by COX5B-UHMK1 axis.

### 2.8. COX5B-UHMK1-ERK Signal Loop Regulates Cell Proliferation and Migration in Hepatoma Cells

**Figure 8:**
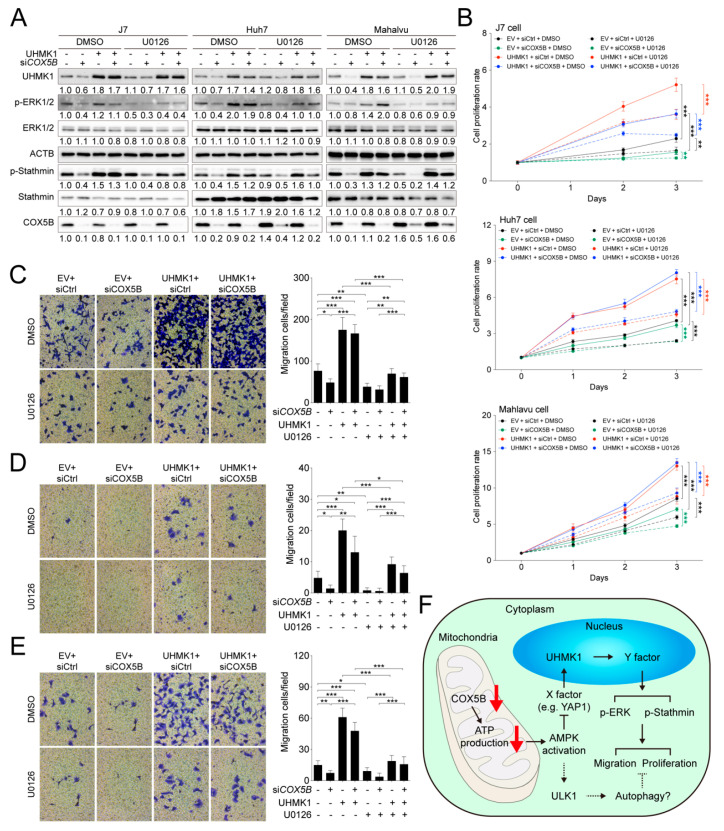
The COX5B-UHMK1-ERK cascade regulates hepatoma cells proliferation and migration. (**A**) The western blot analysis of the phosphorylation status of ERK and Stathmin with or without the treatment of ERK inhibitor U0126 in hepatoma cells. The western blot images in (**E**) were all acquired by using chemStudio PULS imaging system. (**B**) The cell proliferation curves of hepatoma cells receiving the indicated treatments. The transwell-based assays to examine cell migration ability under the indicated treatments in (**C**) J7, (**D**) Huh7 and (**E**) Mahlavu cells (left panel). The quantitative results were shown in right panel. The *p* value was derived from paired two-tail student *t*-test. *, *p* < 0.05; **, *p* < 0.01; ***, *p* < 0.001. All the in vitro cell-based assays shown in this figure were conducted in duplicates. (**F**) The schematic depiction summarizing the key findings of this study. X and Y indicated the unknown factors involved in the regulatory events downstream of COX5B. The dotted lines indicated possible routes affected by COX5B, which remained to be characterized in the future.

To understand whether ERK2 indeed served as one of the modulators downstream of UHMK1 for cell proliferation and migration control, a series of combination experiments including knockdown of COX5B, overexpression of UHMK1 and addition of ERK inhibitor were performed. To alter ERK activity, the inhibitor U0126 was used [25]. As was displayed in Figure 8A, phosphorylation of ERK1/2 was significantly diminished when cells treated with U0126, despite a higher amount of UHMK1 was present in cells. Consequently, it was discovered that the presence of ERK inhibitor partially restrained UHMK1 overexpression-mediated increase of cell proliferation (Figure 8B). Similarly, the migratory ability of hepatoma cells expressing higher levels of UHMK1 was also partially repressed by adding ERK inhibitor (Figure 8C–E), indicating that the ERK pathway was one of the downstream cascades modulated by COX5B-UHMK1 axis for regulation of cell proliferation and migration in hepatoma cells.

In summary, in the present study, the oncogenic role of COX5B in hepatoma was demonstrated. The loss of COX5B-mediated facilitation of bioenergy production resulted in AMPK activation, leading to repression of UHMK1 (an oncogene) expression and induction of ULK1 (a tumor suppressor) via unidentified mechanisms. Lowering UHMK1 expression restrained activation of ERK, which together with the ULK1-induced autophagy, inhibited cell proliferation and migration in hepatoma cells (Figure 8F).

## 3. Discussion

Accumulating evidences over the decades have supported the view that bioenergetic alteration is a crucial factor for development and progression of several types of cancers, including hepatoma [5,6,7,8]. In silico-based and clinical tissue-based experiments, aiming at identification of potential bioenergy modulators controlling hepatoma progression, revealed COX5B. Clinical correlation studies showed that COX5B likely served as a growth-promoting gene and could be used as a predictor for clinical outcomes in hepatoma patients (Figure 1 and Figure S1). As such, mechanistic studies were subsequently conducted. Experimental alterations of COX5B levels revealed that it indeed exhibited a growth-promoting effect, enhancing hepatoma cells proliferation and migration (Figure 2).

In the literature, COX5B has also been proposed as a predictor of clinical outcomes in a various types of cancers, including breast cancer, glioma, gastric cancer, head and neck squamous cell carcinoma (HNSCC) and clear cell renal cell carcinoma (ccRCC) [22,26,27,28,29]. Except for HNSCC, the role of COX5B in almost all other types of cancers exhibited as growth-promoting property as higher levels of COX5B correlated with unfavorable clinical outcomes, similar to what was found in this study. However, the molecular mechanisms of COX5B in modulating cancer growth remained largely unknown.

A huge number of studies had demonstrated that the Warburg effect could benefit tumor progression through shifting bioenergy-generating source to the less time-consuming aerobic glycolysis from OXPHOS [10,11]. However, in this study, silencing COX5B in hepatoma cells was not sufficient to shift the bioenergy-producing origin to glycolysis, except for Huh7 cells, despite it indeed resulted in reduced OXPHOS activity (Figure S5). This observation was consistent with previous findings that silencing COX5B alone was unable to enhance glycolytic capacity but indeed restrained mitochondrial respiration ability, while knockdown of COX4I1, another COX subunit, revealed both effects [30,31]. Additionally, in these studies, the degree of respiration rate declined under silencing COX5B was also not as severe as that of reducing COX4I1, further implying that COX5B might be not as essential as COX4I1 for optimal function of COX.

To dissect the underlying growth-modulating mechanisms downstream of COX5B in hepatoma, UHMK1 (up-regulated) and ULK1 (down-regulated) were identified by microarray analysis followed by transcript-quantification verification (Figure 4 and Figure S3). Expression of UHMK1 was further verified as an event downstream of OXPHOS dysfunction because inactivation of complex I, complex III, complex IV and ATP synthase respectively by chemical inhibitors rotenone, antimycin A, azide and oligomycin all led to reduction of UHMK1 expression (Figure 6A,B and Figure S9), suggesting a potential role of UHMK1 expression for being as a biomarker of OXPHOS dysfunction, at least in hepatoma cells. UHMK1 is a kinase that has been reported as a cell proliferation enhancer by modulating cell cycle progression [32,33] and was thereby considered as an oncogene. As for ULK1, it has been well-studied as a key initiator of autophagy, a self-digestion process for recycling cellular fuel, producing bioenergy and inducing cell death [34] and is therefore believed to be a potent tumor suppressor. Moreover, it was found that the possible regulatory event responsible for the altered expression of UHMK1 and ULK1 might depend on activation of AMPK pathway induced by altered COX5B-mediated bioenergy production (Figure 6 and Figure S6–S8). Although the details and the exact molecule(s) controlling expression levels of UHMK1 and ULK1 downstream of AMPK remained unknown, functional phosphorylation change of ULK1 by activated AMPK has been well-investigated [35] (Figure S8).

Recently, a report demonstrated that *UHMK1* expression was drived by an oncogene called yes-associated protein 1 (YAP1), a key factor in Hippo signaling pathway, through a combination effect of FOXM1 in hepatoma [36,37]. Besides, several studies have illustrated that AMPK-mediated phosphorylation repressed YAP1 activity [38]. As such, it is possible that the COX5B-mediated down-regulation of UHMK1 was a consequence of AMPK-modulated Hippo signaling activation, downstream of bioenergetic alteration. The extent of YAP1 de-phosphorylation may vary among distinct cell lines, while extensive de-phosphorylation may be required to activate YAP1 and thereby promote expression of UHMK1. As such, several different cellular factors capable of affecting YAP1 phosphorylation status could modulate UHMK1 levels. Activation of AMPK directly phosphorylates YAP1 to repress its activity and therefore lowers UHMK1 expression. We speculate that in J7 cells (but not SK-Hep1 cells), AMPK knockdown alone did not adequately reduce YAP1 de-phosphorylation (to activate YAP1) and significantly increase UHMK1. However, when cells were treated by oligomycin/Antimycin A, AMPK was activated (YAP1 was repressed and UHMK1 was already reduced). Therefore, when AMPK was knockdown under oligomycin/Antimycin A treatment, the UHMK1 elevation effects became more prominent (Figure 6E,F and Figure 8F).

Despite a bundle of phospho-proteins that have been proposed as substrates [32,36,39,40,41,42,43,44], it is still unclear regarding the key downstream signaling pathway(s) perturbed by UHMK1 during tumorigenesis and progression. However, by mining the downstream targets of UHMK1 kinase activity, ERK2 and Stathmin1 were identified harboring the most significant changes of phosphorylation status (Table 1). This observation is consistent with previous reports suggesting that Stathmin directly interacted with UHMK1 and served as a protein partner or a substrate for the kinase activity in human and in mice [39,45]. Stathmin plays an essential role to regulate microtubule dynamics and is crucial for cellular processes such as cell division and motility. UHMK1-mediated Stathmin phosphorylation changes likely alter these processes. On the other hand, a systemic examination of the involvement of ERK pathway downstream of COX5B-UHMK1 displayed that the COX5B-UHMK1-ERK signal cascade was indeed crucial for regulation of cell proliferation and migration in hepatoma cells (Figure 8). These findings provide an explanation for the role of UHMK1 in modulating cancer growth, as it has been well-known that activation of ERK pathway plays important roles in tumor growth and migration in several cancers including hepatoma [46].

Upon COX5B silencing, the cells could suffer from various types of stress caused by loss of ATP, but at least not through the oxidative stress caused by ROS, and AMPK was activated to affect UHMK1 expression. The relationship between reduced bioenergy and activation of AMPK upon stress has been well investigated and extensively reviewed. Therefore, activation of AMPK by silencing COX5B, which significantly reduced ATP and increased AMP levels was considered firmly linked. A questionable issue is whether AMPK-mediated regulation of UHMK1 expression is a direct or indirect effect. Although a direct link could not be established, our gene expression perturbing experiments showed an up- and downstream relationship between the COX5B- and UHMK1-mediated regulation of cell growth. As shown in Figure 7, the COX5B-induced proliferation and migration effects could be largely reversed by exogenous expression of UHMK1. In the further downstream, activation of stathmin through UHMK1 has been documented in other previous reports with the capability to promote hepatocarcinogenesis, progression and metastasis by modulating microtubule dynamics [39,45]. Finally, activation of ERK1/2 through UHMK1 may not be totally direct but it indeed could be reversed when ERK1/2 inhibitor was added. Taken together, although COX5B silencing-mediated ATP deficiency could cause other kinds of cell stress, our experimental evidences indicated that the AMPK-UHMK1-ERK/Stathmin cascade constituted a great part in the molecular events so as to inhibit hepatoma cell growth.

## 4. Materials and Methods

### 4.1. Patients and Samples

Three different cohorts of hepatoma patients were analyzed. Firstly, the online available cDNA microarray datasets, GSE63898 and GSE76427, were used for genes expression analyses. Secondly, 102 pairs of paraffin-embedded tumorous and non-tumorous liver tissues were employed for IHC staining. Thirdly, 157 pairs of patients-derived tumorous and non-tumorous liver tissues were utilized for validation by western blotting and real-time quantitative PCR (RT-qPCR). All the paired tissues were obtained from Tissue Bank, Chang Gung Memorial Hospital under the permission of the institutional review board, Chang Gung Memorial Hospital, Linkou, Taiwan (201600084A3).

### 4.2. Immunohistochemistry (IHC) Staining

The IHC staining was executed as described previously [21]. The retrieved surgically resected tumorous and adjacent non-tumorous tissues from our tissue bank were used for immunohistochemistry (IHC) staining. Deparaffinized rehydrated sections were treated with H_2_O_2_ (3% in distilled water) for 15 min, pre-incubated with normal serum (goat serum for rabbit-derived antibodies as primary antibodies and horse serum for mouse-derived antibodies as primary antibodies) in phosphate-buffered saline (PBS) with the dilution of 1:10 for 40 min. Subsequently, it was incubated with the specific primary antibodies for 1 h at room temperature. After washed by PBS for three times, 5 min each, the sections were then incubated in PBS with biotinylated secondary antibodies [anti-rabbit immunoglobulin G (IgG) (Vector Lab, Burlingame, CA, USA, BA-1000) for rabbit-derived antibodies and anti-mouse IgG (Vector Lab, BA-9200 for mouse-derived antibodies] in 1:400 dilution at room temperature for 40 min. To conjugate horseradish peroxidase (HRP), the VECTASTAIN Elite ABC HRP kit (Vector Lab, PK-6100) was used following the protocol provided by manufacturer. Visualization was performed by using the 3,3’-diaminobenzidine substrate kit (Vector Lab, SK-4100). The details of primary antibodies employed in this study for IHC were given as below: rabbit anti-COX5B (1:500) (Abcam, Cambridge, UK, ab180136); mouse anti-UHMK1 (1:200) (GeneTex, Irvine, CA, USA, GTX83440); rabbit anti-AMPKα1 (1:100) (Proteintech, Rosemont, IL, USA, 10929-2-AP); rabbit anti-AMPKα1 Thr172 (1:100) (Abclonal, Woburn, MA, USA, AP0116); rabbit anti-AMPKα1 Ser485 (1:100) (Abclonal, AP0871); rabbit anti-ULK1 (1:100) (Proteintech, 20986-1-AP); rabbit anti-ULK1 Ser555 (1:100) (Abclonal, AP0760); rabbit anti-ULK1 Ser423 (1:100) (Abclonal, AP0654); rabbit anti-ULK1 Ser757 (1:100) (Abclonal, AP0736). The IHC intensities were separately examined by two experienced pathologists. The IHC intensities were obtained by using ImageJ software following procedures described in a previous report [47]. The difference in COX5B staining intensities in the tumorous and nontumorous parts, tumor > nontumor or tumor ≤ nontumor, was used to categorize patients for survival curves analyses.

### 4.3. Lysate Preparation and Western Blot

Protein extraction and western blot analysis were conducted as described previously [48]. In brief, the protein lysates were extracted by adding a sufficient volume of radio-immunoprecipitation assay (RIPA) buffer [20 mM Tris-HCl (pH 7.5), 150 mM NaCl, 1 mM Na_2_EDTA, 1 mM EGTA, 1% NP-40] containing 1× protease inhibitor cocktail (BIOTOOLS, New Taipei city, Taiwan, TAAR-BBI2) to the collected cells in tubes. The tubes were placed in the 4 °C cold room with constant rotation for 30 min. Subsequently, the mixtures were subjected for centrifuging at 13,000 rpm for 15 min. The protein samples were then separated by 10–12% Bis-Tris polyacrylamide gels and transferred onto PVDF membranes for western blotting. The transferred membranes were then blocked by 5% milk before addition of primary and secondary antibodies. The details of primary antibodies employed in this study for western blotting were: rabbit anti-ACTB (1:20,000) (Proteintech, 60008-1-Ig); rabbit anti-COX5B (1:30,000) (Abcam, ab180136); mouse anti-UHMK1 (1:1000) (GeneTex, GTX83440); rabbit anti-AMPKα1 (1:500) (Proteintech, 10929-2-AP); rabbit anti-AMPKα1 Thr172 (1:500) (Abclonal, AP0116); rabbit anti-AMPKα1 Ser485 (1:500) (Abclonal, AP0871); rabbit anti-ULK1 (1:1000) (Proteintech, 20986-1-AP); rabbit anti-ULK1 Ser555 (1:1000) (Abclonal, AP0760); rabbit anti-ULK1 Ser423 (1:1000) (Abclonal, AP0654); rabbit anti-ULK1 Ser757 (1:1000) (Abclonal, AP0736); rabbit anti-p21 (1:1000) (Proteintech, 10355-1-AP). The details of secondary antibodies employed in this study for western blotting were: HRP-conjugated anti-rabbit (211-032-171, Jackson ImmunoResearch Labs, West Grove, PA, USA, 1:10,000 dilution) or anti-mouse (115-035-174, Jackson ImmunoResearch Labs, 1:10,000 dilution) light-chain-specific secondary antibodies were used for signal detection using WesternBright ECL HRP substrate (Advansta, San Jose, CA, USA, K-12045). Images were acquired by exposing the luminescent signals to X-ray films or by using UVP chemStudio PLUS imaging system. Densitometric analyses of protein signals were achieved by using imageJ software, version 2.

### 4.4. Plasmid Construction and Preparation for Expression

The expressing vector for COX5B-myc-DDK was purchased from Origene, Rockville, MD, USA (RC202511). To overexpress UHMK1, its open reading frame (ORF) was inserted into an expression-competent vector. The primers, 5′-CCCAAGCTTATGGCGGGATCCGGCTGCG-3′ and 5′-CGGGGTACCTTAAAGCAAGGTTTGATACAGAT-3′, were used to amplify UHMK1 ORF. The created *HindIII* and *KpnI* sites were used to digest amplicon and pcDNA3.1/V5-His vector (Invitrogen, Carlsbad, CA, USA, V81020) for subsequent insertion. All constructed plasmids were sequence-verified.

### 4.5. Cell Culture and Transfection

The cells used in this study, J7, SK-Hep1, Mahlavu and Huh7, were cultured in Dulbecco’s Modified Eagle Medium (DMEM) in a standardized culture condition, 5% CO_2_ in a humidified 37 °C incubator. All the cell lines were gifts from Dr. Kwang-Huei Lin’s lab (Chang Gung University, Taoyuan, Taiwan). All the cells used in this study were routinely examined for mycoplasma contamination. For transient expression of COX5B and/or UHMK1, the cells were seeded 16 h before transfection. Five μg of plasmid DNA was used for transfection in a 6-cm plate. The TransIT-LT1 transfection reagent (Mirus, Madison, WI, USA, MIR2300) was used for transfection, according to the procedures provided by the manufacturer. The plasmid transfection efficiencies were ~60% in Huh7 and SK-Hep1 cells, ~40% in J7 cell and ~30% in Mahlavu cell. For knockdown of COX5B, the smart pool siRNAs (Dharmacon, Lafayette, CO, USA, M-013632), siRNA 1: CGACUGGGUUGGAGAGGGA, siRNA 2: GAGCACCUGCACUAAAUUA, siRNA 3: GGGACUGGACCCAUACAAU and siRNA 4: GAGAA UAGUAGGCUGCAUC, were used. The non-targeting pool included four independent scramble siRNAs, UGGUUUACAUGUCGACUAA, UGGUUUACAUGUUGUGUGA, UGGUUUACAUG UUUUCUGA and UGGUUUACAUGUUUUCCUA, was used as control siRNA (Dharmacon, D0018101020). To maximize the transfection efficiency of siRNA, the Lipofectamine^TM^ RNAiMAX transfection reagent (Invitrogen, 13778) was employed according to the protocol provided by the manufacturer.

### 4.6. Lentivirus-Mediated Knockdown of COX5B and AMPKα1

To achieve stable knockdown, the lentivirus-mediated transduction of shRNA against COX5B or AMPK was employed as previously described [49]. The target sequences of shRNA clones used for silencing COX5B was GCATCTGTGAAGAGGACAATA (clone ID: TRCN0000046270) and utilized for downregulating AMPKα1 were CCTGGAAGTCACACAATAGAA (#1, clone ID: TRCN0000000859) and GAAGGTTGTAAACCCATATTA (#2, clone ID: TRCN0000219690). The control used in this assay was shRNA against LacZ, GCCGTCGTATTACAACGTCGT (clone ID: TRCN0000231700). All the shRNA clones were purchased from RNAi core in Academia Sinica, Taipei, Taiwan.

### 4.7. Senescence Beta-Galactosidase Cell Staining Assay

To assess whether the cells under changed COX5B expression levels manifested an altered cell senescence status, the senescence beta-galactosidase cell staining assay (Cell Signaling Technology, Danvers, MA, USA, #9860) was employed according to the protocol provided by the manufacture.

### 4.8. Apoptosis Detection Assay

The apoptosis status of cells under altered COX5B expression were assessed by using the DeadEnd Fluorometric TUNEL System (Promega, Madison, MA, USA, G3250) according to the protocol provided by manufacturer.

### 4.9. Oligomycin, Antimycin A, H_2_O_2_ and U0126 Treatment

To induce bioenergetic alteration, around 80% confluent cells were treated with 1 μM oligomycin (Cell Signaling Technology, #9996) or 2 μg/mL antimycin A (Sigma, St. Louis, MO, USA, A8674) for 48 h and then collected for subsequent analyses. To accumulate ROS, cells were treated by 200 μM H_2_O_2_ (Sigma, 18304) and harvested 6 h later. For repressing the activity of ERK pathway, cells were treated with 10 μM U0126 (Promega, Madison, MA, USA, V112A) and incubated for 48 h before further analysis.

### 4.10. Cell Proliferation Assay

The cell proliferation rate was assessed as previously described [49]. Briefly, for each replicate, 3 × 10^3^ cells were seeded in each well of a 96-well plate. After 24 h post seeded, the Alamar Blue cell viability reagent (Invitrogen, DAL1100) was directly added into culture medium 3 h before cells were harvested for quantifying the fluorescence of metabolite for the results of day 1. Then, the quantification was conducted every day until day 4.

### 4.11. Cell Migration Assay

The transwell assay was used for assessing the cell migration ability. The procedures were conducted as previously described [50]. Briefly, 2–8 × 10^4^ cells were grown in the transwell upper wells (Corning, Corning, NY, USA, #3422). After 24- or 48-h of incubation, the cells on the transwell were stained and counted for quantification.

### 4.12. ROS, ATP and AMP Detection Assays

For quantifying the cellular reactive oxygen species (ROS) and ATP levels, the detection kits (BioVision, Milpitas, CA, USA, K936 for ROS and K354 for ATP) were employed and the assays were conducted according to the protocols provided by manufacturers. For detection of AMP, the AMP-Glo assay (Promega, V5011) was used and the assay was performed following the procedure provided by manufacturer.

### 4.13. Cytochrome c Oxidase Activity Detection Assay

To measure the activity of cytochrome *c* oxidase under depletion of COX5B in hepatoma, the cytochrome *c* oxidase assay kit (Sigma, CYTOCOX1) was utilized and the experiments were conducted according to the instruction provided by the manufacturer. Totally 20–100 μg mitochondrial lysates were subjected to this assay. Mitochondrial proteins were isolated using mitochondria isolation kit (Sigma, MITOISO2) following the manufacturer’s protocols.

### 4.14. Xenograft Model

The xenograft model was executed under the approval of Chang Gung Institutional Animal Care and Use Committee (Approval number: 2015122310). Three-week-old BALB/c nude mice purchased from the National Laboratory Animal Center (Taipei, Taiwan) were used. The procedure was described previously [49]. Briefly, 1 × 10^6^ cells treated by lentivirus-mediated stably silencing of COX5B were resuspended in PBS and were subcutaneously injected into the back of mice. The mice were sacrificed and the tumors were isolated 3–6 weeks post injection.

### 4.15. cDNA Microarray

The cDNA microarray was used for detection of differentially expressed genes and was conducted as previously described [51]. To search for differentially expressed genes under COX5B knockdown, log2 fold change >0.5 and <−0.5 was used as the cutoff criteria.

### 4.16. RNA Isolation and Quantitative RT-PCR

The RNA extraction was performed as previously described [52]. The ToolScript MMLV RTase (TOOLS, New Taipei city, Taiwan, TGERA04) was utilized for reverse transcription. Prior to reverse transcription, the DNase digestion was conducted using Turbo DNase I (Ambion, Austin, TX, USA, AM1907). Ten μg of total RNA was used for each digestion experiment. The experiments were conducted according to the standard protocols provided by the manufacturers. Subsequently, 5 μg of DNA-free RNAs were subjected to RT reaction using ToolScript MMLV RTase (TOOLS, New Taipei city, Taiwan, TGERA04). In each aliquot of 20-μL reaction, 5 μL of 50-fold diluted cDNAs were utilized for RT-qPCR analysis. The Bio-rad CFX96 real time system was used for detection of the genes in interest. The primers used for quantitative RT-PCR in this study was listed as below. ACTB qPCR Forward: CACCAACTGGGACGACATGG; ACTB qPCR Reverse: AGGATCTTCAGAGGTAGTC; UHMK1 qPCR Forward: ACGCTGTCTGTTGCTTGAACT; UHMK1 qPCR Reverse: GGCACAATGCTGTATCATCCAC; ULK1 qPCR Forward: CAGACAGCCTGAT GTGCAGT; ULK1 qPCR Reverse: CAGGGTGGGGATGGAGAT.

### 4.17. Phospho-Peptide Enrichment Analysis

To analyze the phosphorylation status under specific treatment, the phospho-peptide enrichment assay was conducted. Briefly, the trypsinized protein samples were labeled with iTRAQ reagent. After pooling and desalting of the peptides, they were bound with TiO_2_ for phospho-peptide enrichment. The enriched phospho-peptides were subsequently identified by LC/MS/MS. For LC/MS/MS, the dried peptide mixtures were reconstituted in HPLC buffer A (0.1% formic acid) and loaded onto a reverse-phase column (Zorbax 300SB-C18, 0.3 × 5 mm; Agilent Technologies, Santa Clara, CA, USA). The desalted peptides were then separated on a column (HydroRP 2.5 μm, 75 μm I.D. × 20 cm with a 15 μm tip) using a multi-step gradient of HPLC buffer B (99.9% acetonitrile/0.1% formic acid) for 120 min with a flow rate of 0.25 μL/min. The LC apparatus was coupled with a 2D linear ion trap mass spectrometer (Orbitrap Elite; Thermo Fisher Scientific, Waltham, MA, USA) operated using Xcalibur 2.2 software (Thermo Fisher). The full-scan MS was performed in the Orbitrap over a range of 400 to 2000 Da and a resolution of 120,000 at m/z 400. The 16 data-dependent MS/MS scan events (8 CID MS^2^, 8 HCD) were followed by one MS scan for the eight most abundant precursor ions in the preview MS scan. The m/z values selected for MS/MS were dynamically excluded for 80 s with a relative mass window of 15 ppm. The electrospray voltage was set to 2.0 kV, and the temperature of the capillary was set to 200 °C. MS and MS/MS automatic gain control were set to 1000 ms (full scan) and 200 ms (MS/MS), or 3 × 10^6^ ions (full scan) and 3 × 10^3^ ions (MS/MS) for maximum accumulated time or ions, respectively.

### 4.18. Statistical Methods

Parametric data in normal distribution were presented as mean ± standard deviation and compared by student’s *t*-test. Survival analysis was performed by Kaplan-Meier analysis. Patients were divided into subgroups possessing high and low levels of variable(s) for prognostic analyses. The cutoff was determined by use of the minimal *p* value approaches for clinical studies [53].

## 5. Conclusions

In the present study, it was found that COX5B was a master gene to control a signaling cascade composed of AMPK-UHMK1-ERK, operating through COX5B-mediated bioenergetic alterations. Although the molecular details of the linkages between molecules in this axis require further investigation, identification of this cascade maybe informative for development of novel therapeutic strategies for treatment of hepatoma. Additionally, these findings shed light on a fundamental issue in cancer biology, explaining how bioenergetic alteration controls tumorigenesis.

## Figures and Tables

**Table 1:** The candidates of UHMK1 downstream phosphorylated effector.

Gene Name	Localization	Sequence	Position	Site	Ratio 1 ^a^	Ratio 2 ^b^	Ratio 3 ^c^
NPM1	Nuclear	DELHIVEAEAMNYEGSPIK	55–73	S70	0.8	1.3	1.9
MYH10	Cytoplasmic	QLHLEGASLELSDDDTESK	1976–1994	S1987	0.8	1.2	1.3
C11orf58	Cytoplasmic	RSASPDDDLGSSNWEAADLGNEER	14–37	S17	0.8	1.3	1.7
ERK2	Cytoplasmic	VADPDHDHTGFLTEYVATR	190–208	Y204	0.5	1.8	3.5
Stathmin1	Cytoplasmic	SKESVPEFPLSPPK	28–41	S38	0.7	1.3	1.4
ZC3H13	Nuclear	GNIETTSEDGQVFSPK	980–995	S993	0.8	1.3	1.3
DYNC1LI2	Cytoplasmic	DFQDYMEPEEGCQGSPQRR	180–198	S194	0.9	1.4	1.1
STIP1	Cytoplasmic	HDSPEDVK	526–533	S528	0.8	1.1	1.1
FIP1L1	Nuclear	ERDHSPTPSVFNSDEER	488–504	S492	0.8	1.1	1.2
HIRIP3	Nuclear	TLDSDEERPRPAPPDWSHMR	527–546	S530	0.9	1.1	1.1
TCEA1	Nuclear and cytoplasmic	EPAITSQNSPEAR	92–104	S100	0.7	1.2	1.3
MYBBP1A	Nuclear	DPAQPMSPGEATQSGARPADR	5–25	S11	0.8	1.2	1.2

^a^ The ratio was derived by normalizing the counts from cells with empty vector plus COX5B knockdown to counts from cells with empty vector plus control knockdown. ^b^ The ratio was derived by normalizing the counts from cells with UHMK1 overexpression plus control knockdown to counts from cells with empty vector plus control knockdown. ^c^ The ratio was derived by normalizing the counts from cells with UHMK1 overexpression plus COX5B knockdown to counts from cells with empty vector plus COX5B knockdown.

## Supplementary Materials

The following are available online at https://www.mdpi.com/2072-6694/12/6/1646/s1, Figure S1: Survival rate analysis of COX subunits to examine their prognostic roles in HCC, Figure. S2: Silencing COX5B reduces classical migration marker Snail expression. Figure S3: COX5B affects ULK1 expression in HCC, Figure S4: The Kaplan-Meyer analysis of prognosis in HCC patients stratified by the T/N ratio of *COX5B* and *UHMK1* mRNA levels, Figure S5: Silencing COX5B impacts OXPHOS but has limited effects on shifting the bioenergy metabolism to aerobic glycolysis, Figure S6: Bioenergetic alteration orchestrates ULK1 expression in HCC cells, Figure S7: Depletion of COX5B induces activation of AMPK, Figure S8: Bioenergy alteration mediated promotion of ULK1 expression is AMPK-activation-dependent, Figure S9: UHMK1 expression is affected by inhibitors of OXPHOS complex I and IV.

## Abbreviations

AMP	adenosine monophosphate
AMPK	AMP-activated protein kinase
COX	cytochrome *c* oxidase
COX5B	Cytochrome *c* oxidase subunit 5B
IHC	immunohistochemistry
MFS	metastasis-free survival
OS	overall survival
OXPHOS	oxidative phosphorylation
RFS	recurrence-free survival
ROS	reactive oxygen species
RT-qPCR	real-time quantitative PCR
UHMK1	U2AF homology motif kinase1
ULK1	Unc-51 like autophagy activating kinase 1
