# Supplementary material for: COX5B-Mediated Bioenergetic Alteration Regulates Tumor Growth and Migration by Modulating AMPK-UHMK1-ERK Cascade in Hepatoma
Source: Cancers (Basel). 2020 Jun 22;12(6):1646. doi: 10.3390/cancers12061646 (PMC7352820; doi:10.3390/cancers12061646)

Supplementary Materials: COX5B-Mediated Bioenergetic Alteration Regulates Tumor Growth and Migration by Modulating AMPK-UHMK1-ERK Cascade in Hepatoma

Yu-De Chu [1,†], Wey-Ran Lin [1,2,3,†], Yang-Hsiang Lin [1], Wen-Hsin Kuo [1], Chin-Ju Tseng [3], Siew-Na Lim [3,4], Yen-Lin Huang [5], Shih-Chiang Huang [5], Ting-Jung Wu [1], Kwang-Huei Lin [1] and Chau-Ting Yeh [1,2,3,6,*]

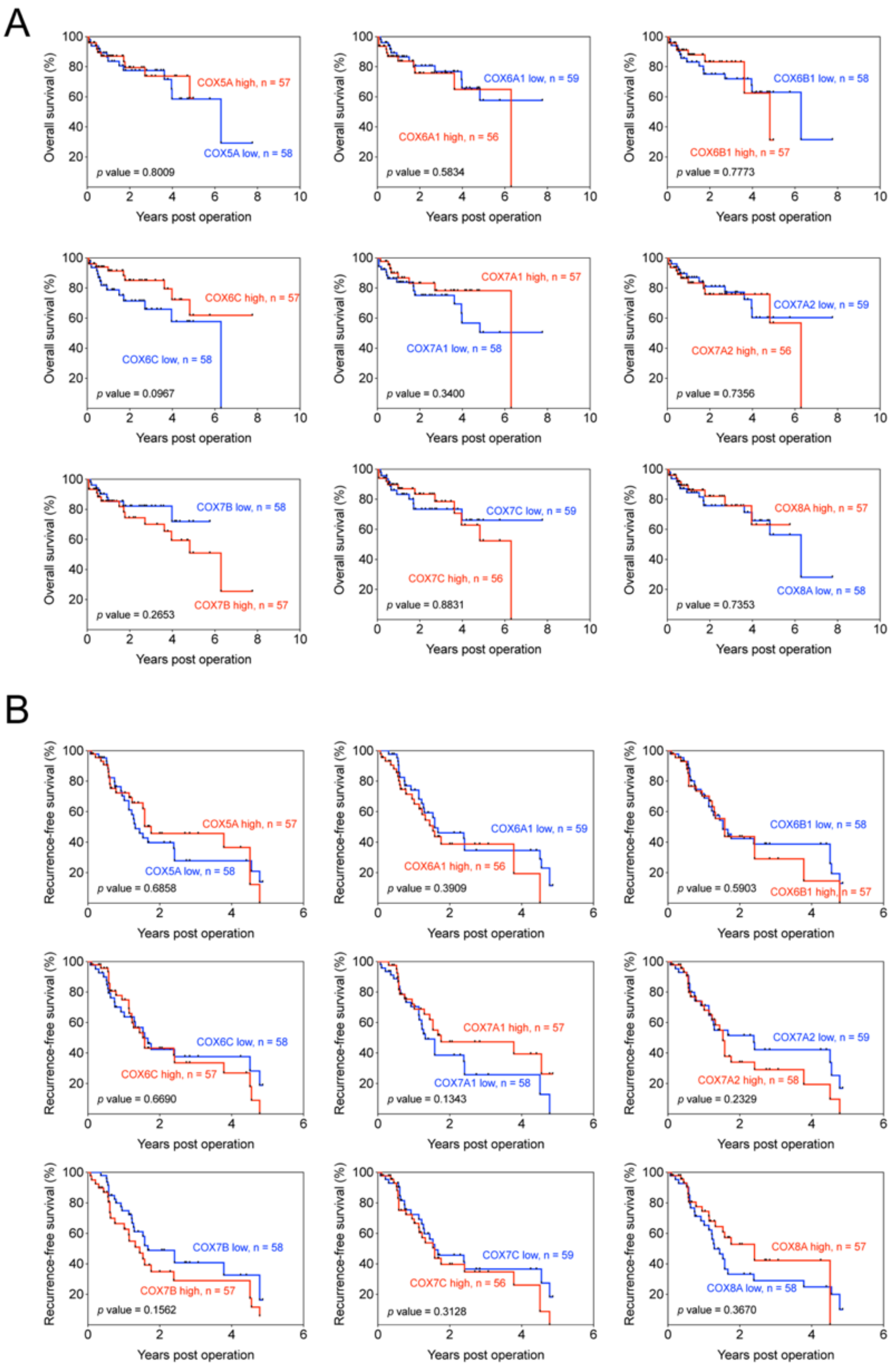

Figure S1. Survival rate analysis of COX subunits to examine their prognostic roles in HCC**.** This figure was used to supplement Figure 1A-B in the main text. The results of Kaplan-Meyer analysis for (**A**) overall survival and (**B**) recurrence-free survival in HCC patients from GSE76427 dataset were depicted. The patients were grouped according to the mean of transcript levels.

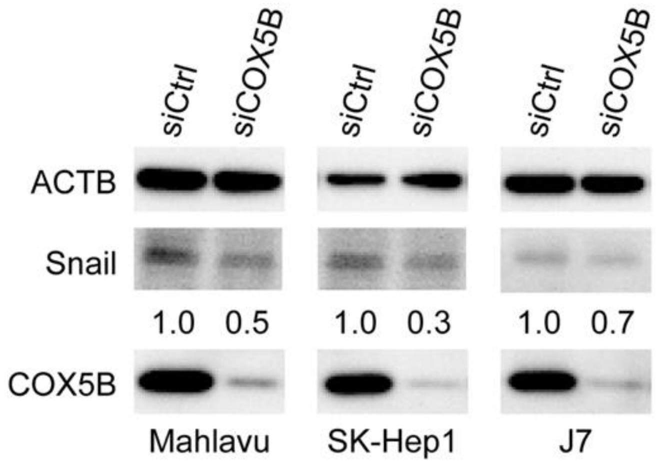

Figure S2. Silencing COX5B reduces classical migration marker Snail expression. This figure was used to supplement Figure 2C. Western blot analysis of samples from indicated hepatoma cells with or without knockdown of COX5B. Snail was used as a marker of migration.

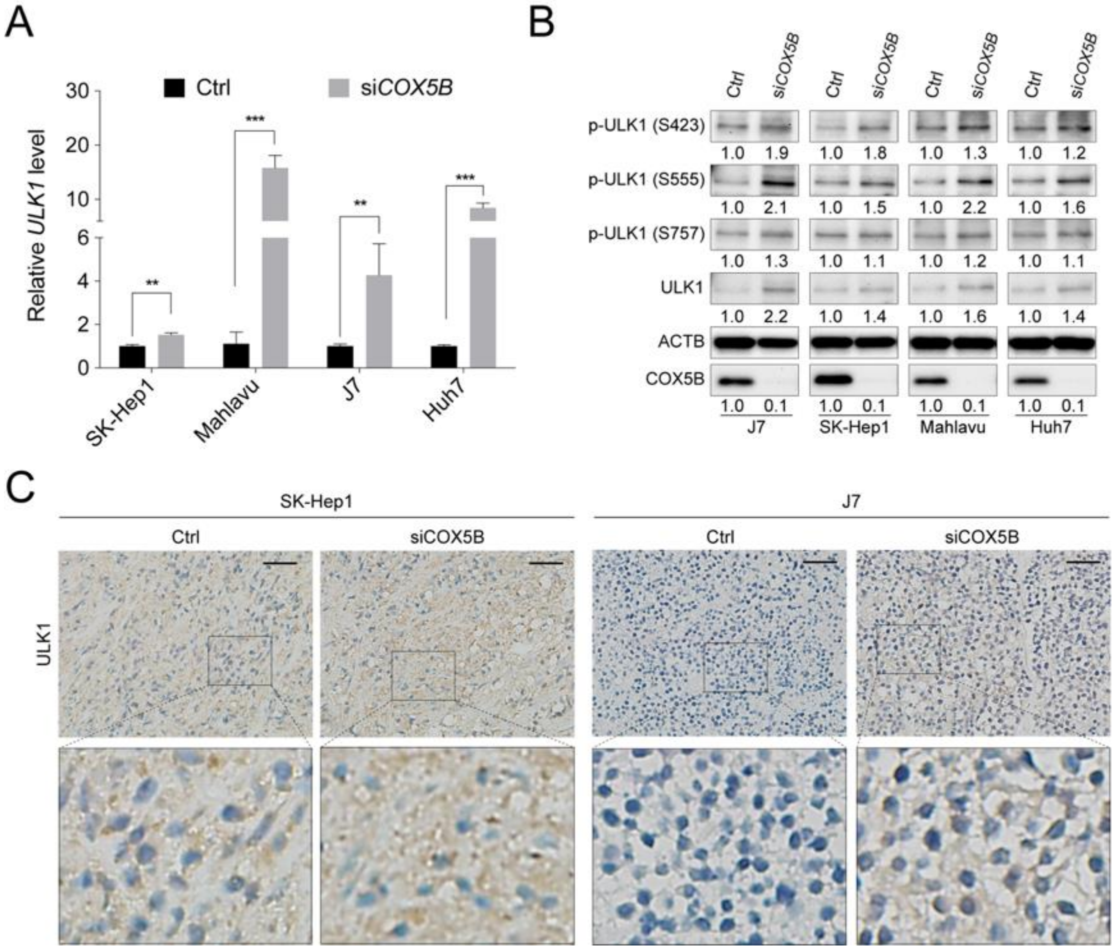

Figure S3. COX5B affects ULK1 expression in HCC. This figure was used to supplement Figure 4 in the main text. (**A**) RT-qPCR and (**B**) western blot were performed to validate ULK1 level elevations after depletion of COX5B. (**C**) IHC staining of paraffin-embedded tissues derived from Figure 6E. The black bar represented a scale bar of 50 μm.

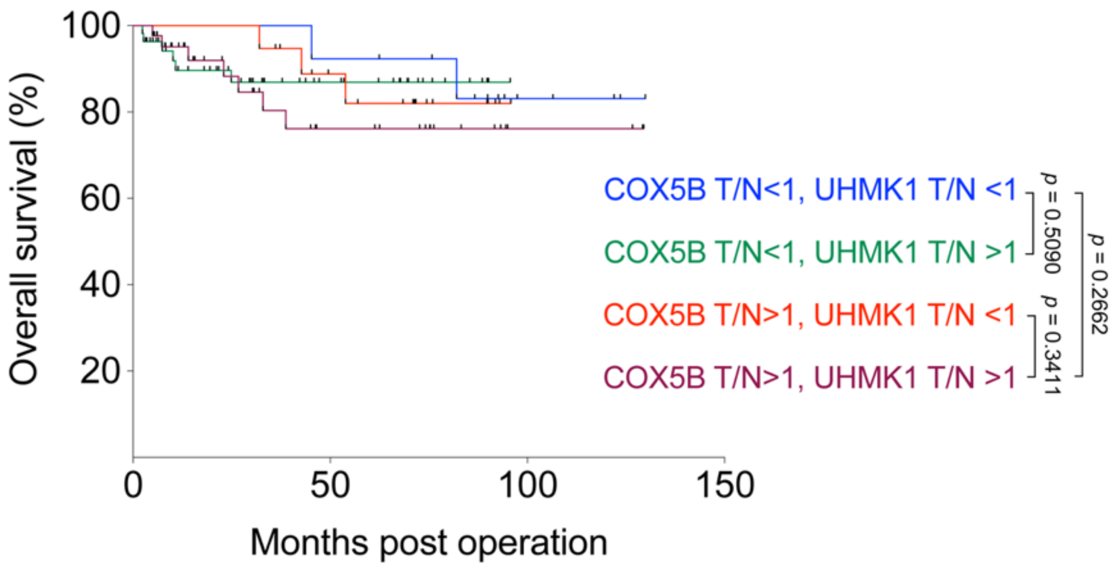

Figure S4. The Kaplan-Meyer analysis of prognosis in HCC patients stratified by the T/N ratio of *COX5B* and *UHMK1* mRNA levels. This figure was used to supplement Figure 4F in main text.

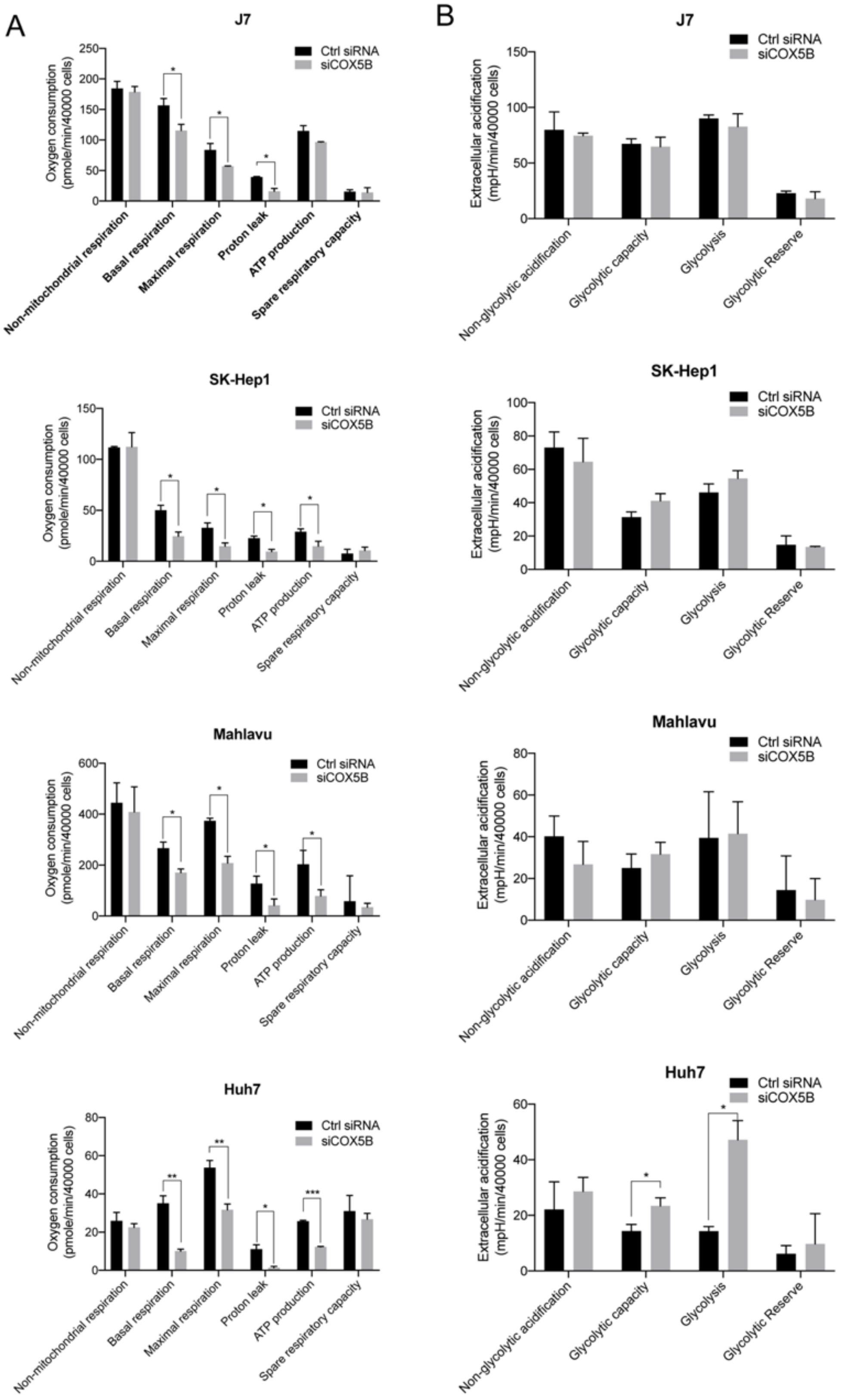

Figure S5. Silencing COX5B impacts OXPHOS but has limited effects on shifting the bioenergy metabolism to aerobic glycolysis. The seahorse analysis was performed using indicated hepatoma cells with or without silencing of COX5B. The p value was derived from paired two-tail student t-test. *, $p < 0.05$; **, $p < 0.01$; ***, $p < 0.001$.

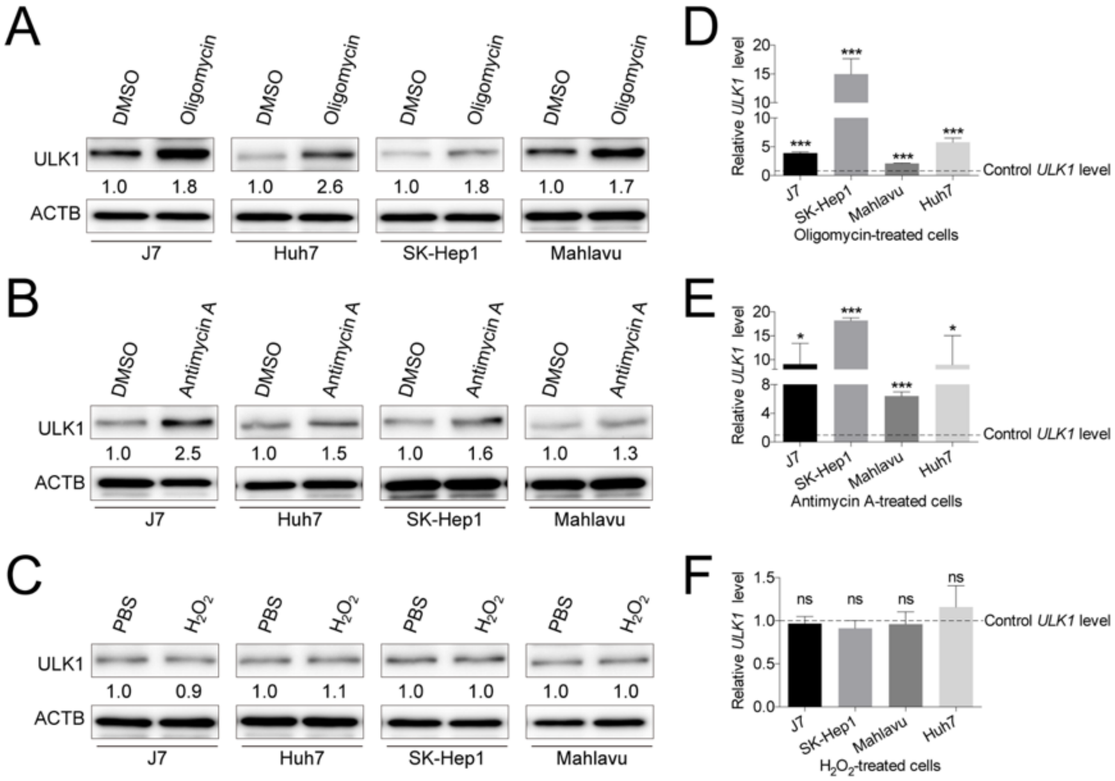

Figure S6. Bioenergetic alteration orchestrates ULK1 expression in HCC cells. This figure was intended to supplement Figure 6D in the main text. Western blot and RT-qPCR analysis of cell lysates derived from cells treated with chemical drugs. (**A and D**) Oligomycin and (**B and E**) antimycin A were used to block the bioenergy production. (**C and F**) H_2O_2 was used for the purpose of accumulating ROS. The *p* value was derived from paired two-tail student *t*-test. *, $p < 0.05$; **, $p < 0.01$; ***, $p < 0.001$. ns, not significant.

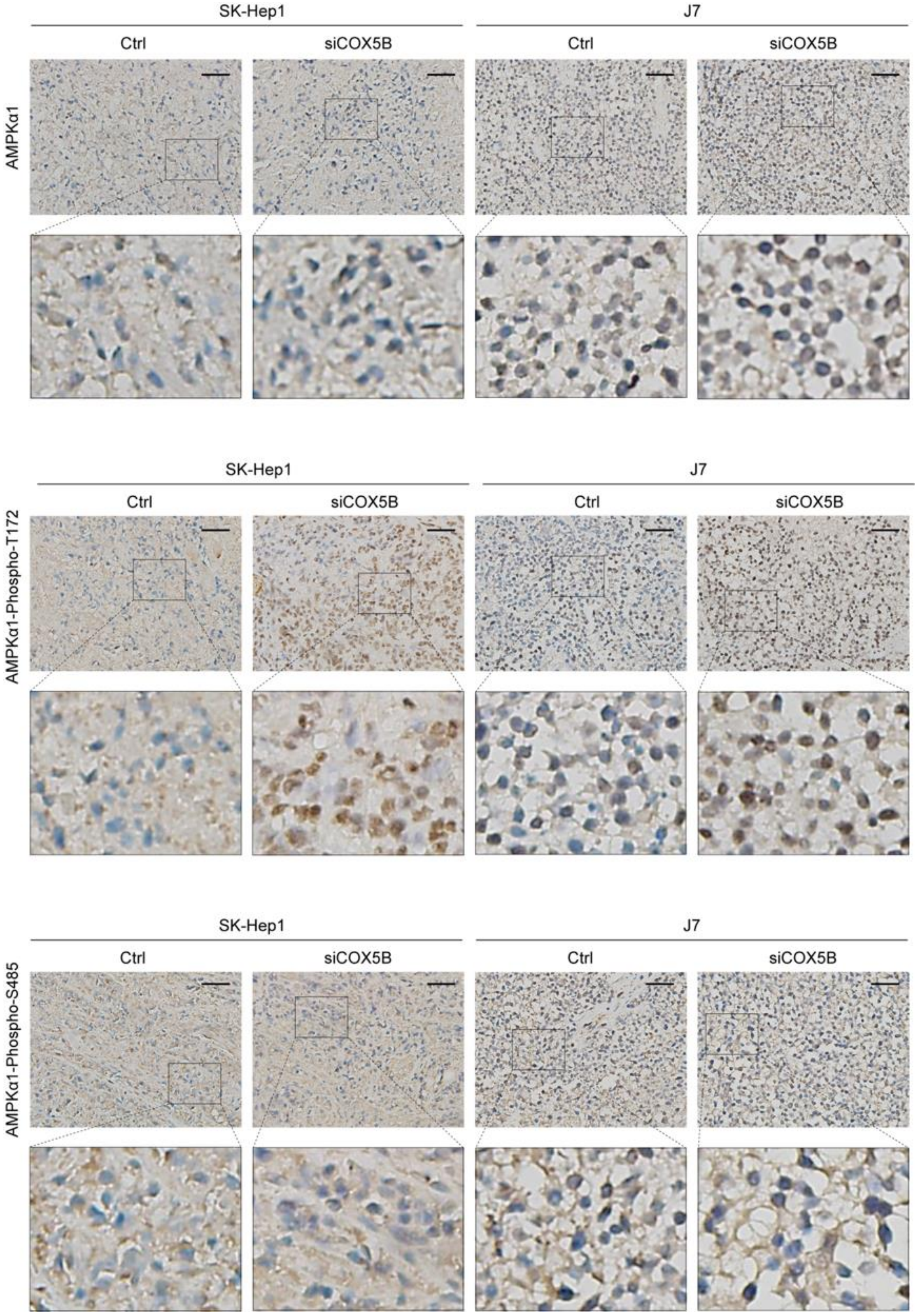

Figure S7. Depletion of COX5B induces activation of AMPK. This figure was intended to supplement Figure 6D in the main text. The IHC staining of paraffin-embedded tissues derived from Figure 2E was performed by using the indicated antibodies. The black bar represented a scale bar of 50 μm.

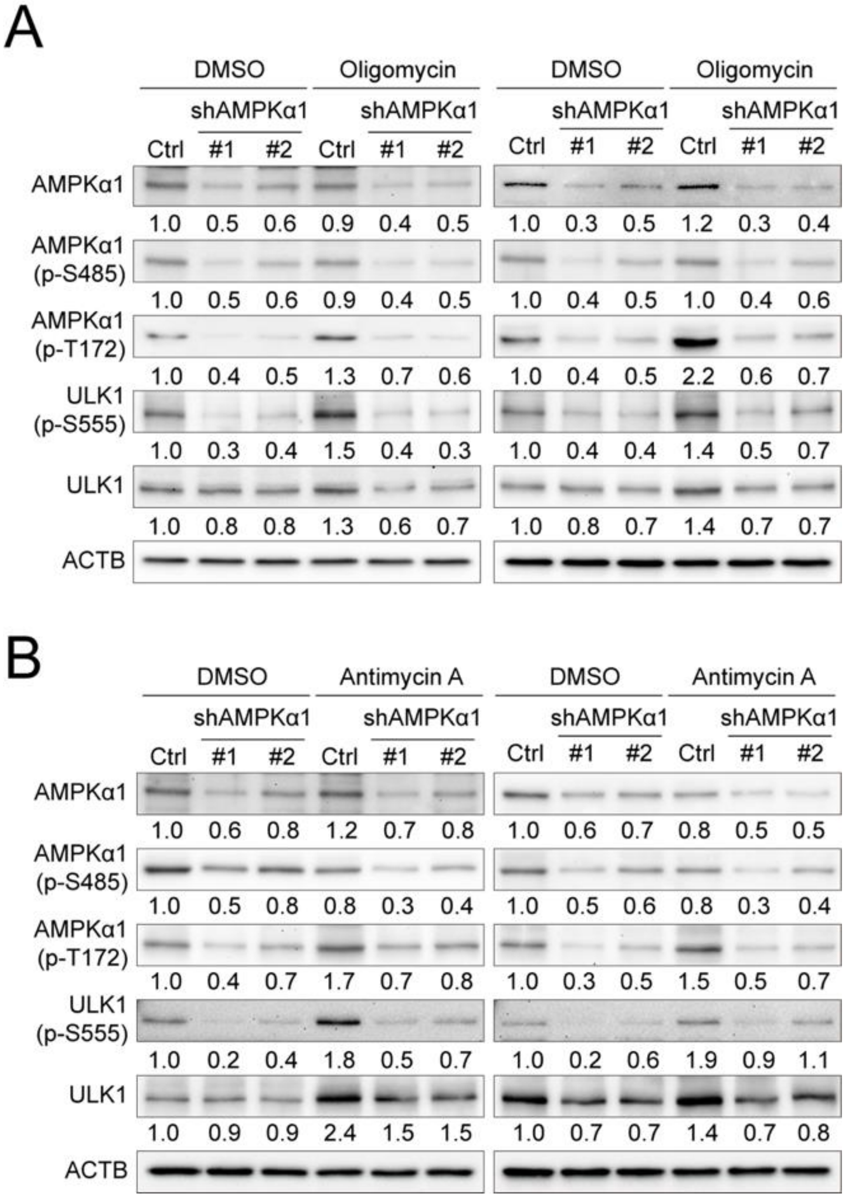

Figure S8. Bioenergy alteration mediated promotion of ULK1 expression is AMPK-activation-dependent. This figure was used to supplement Figure 6E and F in the main text. Western blot showed the results of combined analysis for the relationship between bioenergy alteration, induced by (**A**) oligomycin and (**B**) antimycin A, and activation of AMPK.

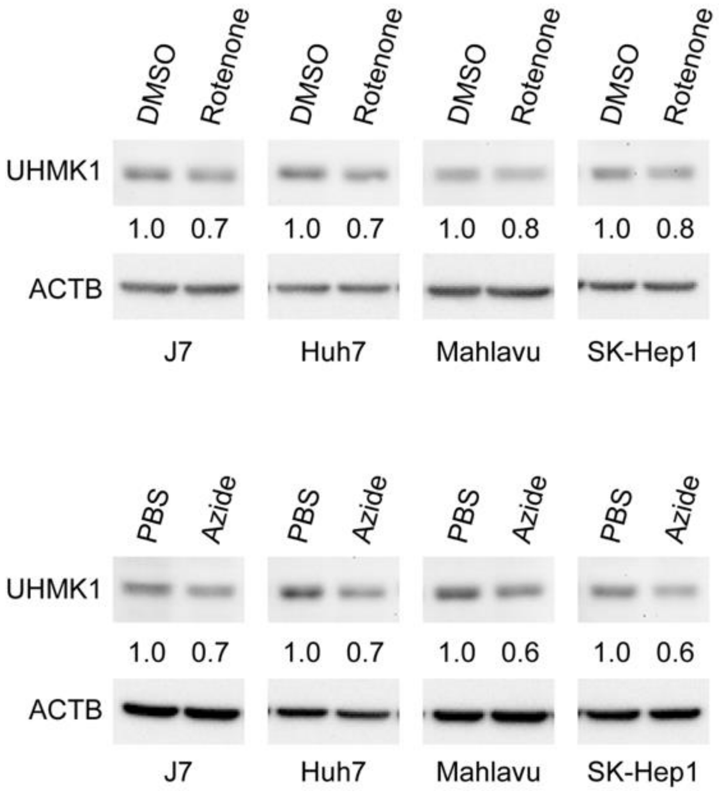

Figure S9. UHMK1 expression is affected by inhibitors of OXPHOS complex I and IV. This figure was used for the discussion part. Western blot analysis of samples from indicated hepatoma cells treated with or without chemical inhibitors. Rotenone was used to inhibit OXPHOS complex I while azide was used to repress complex IV.

The western blot raw images for Figure 1E

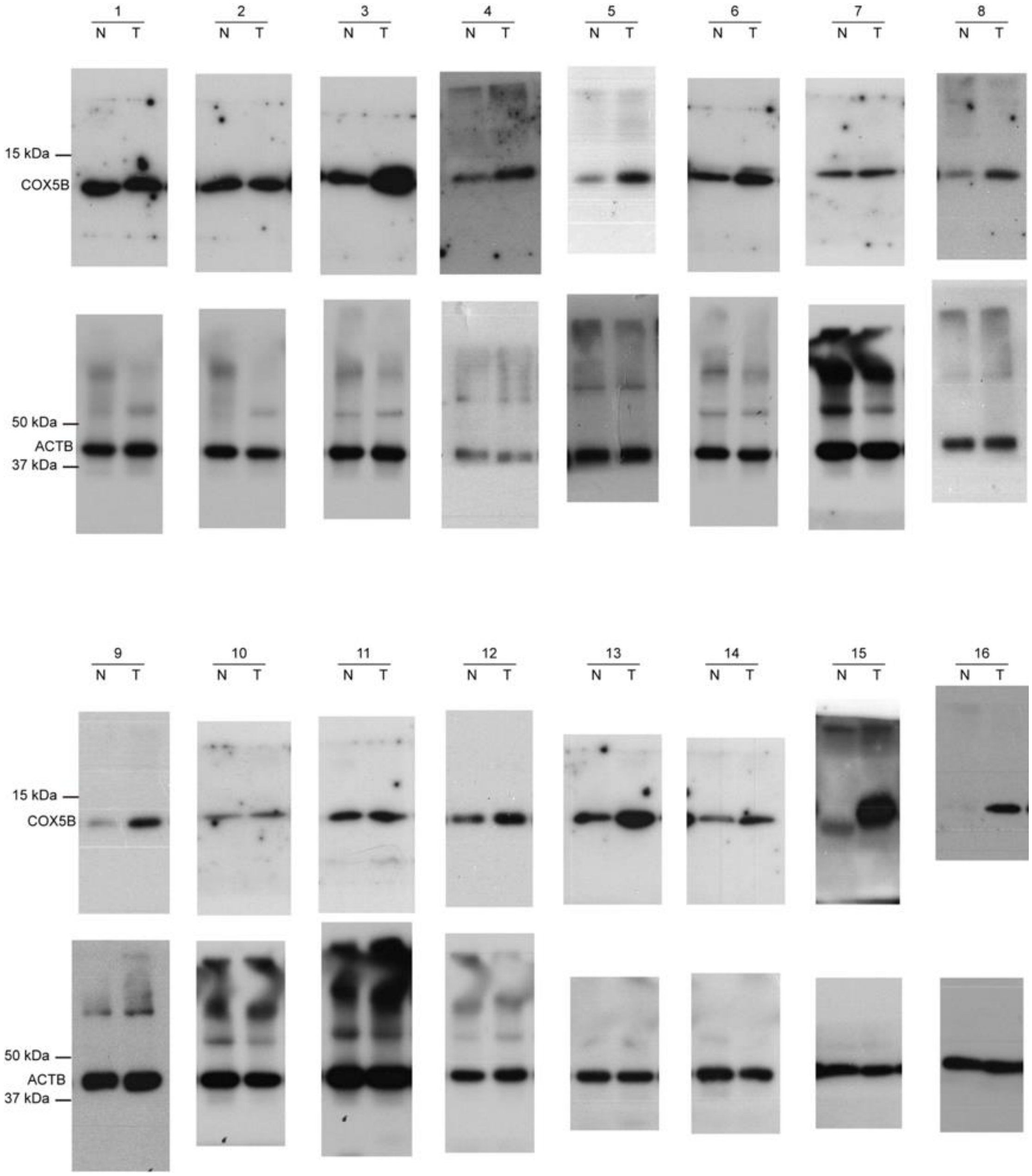

The western blot raw images for Figure 1E

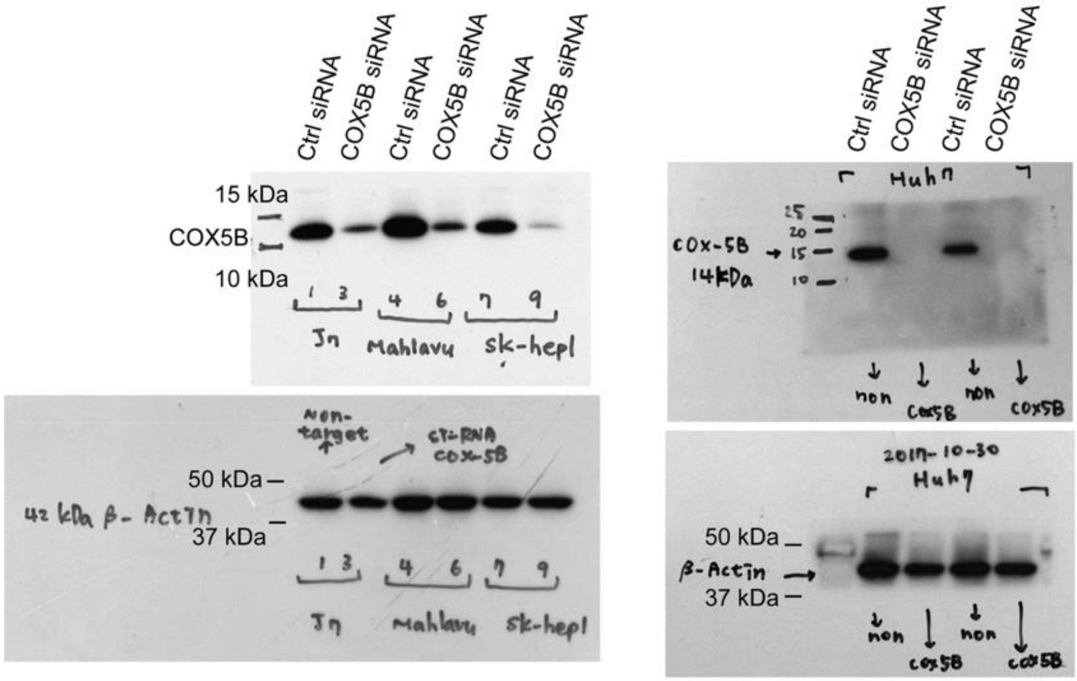

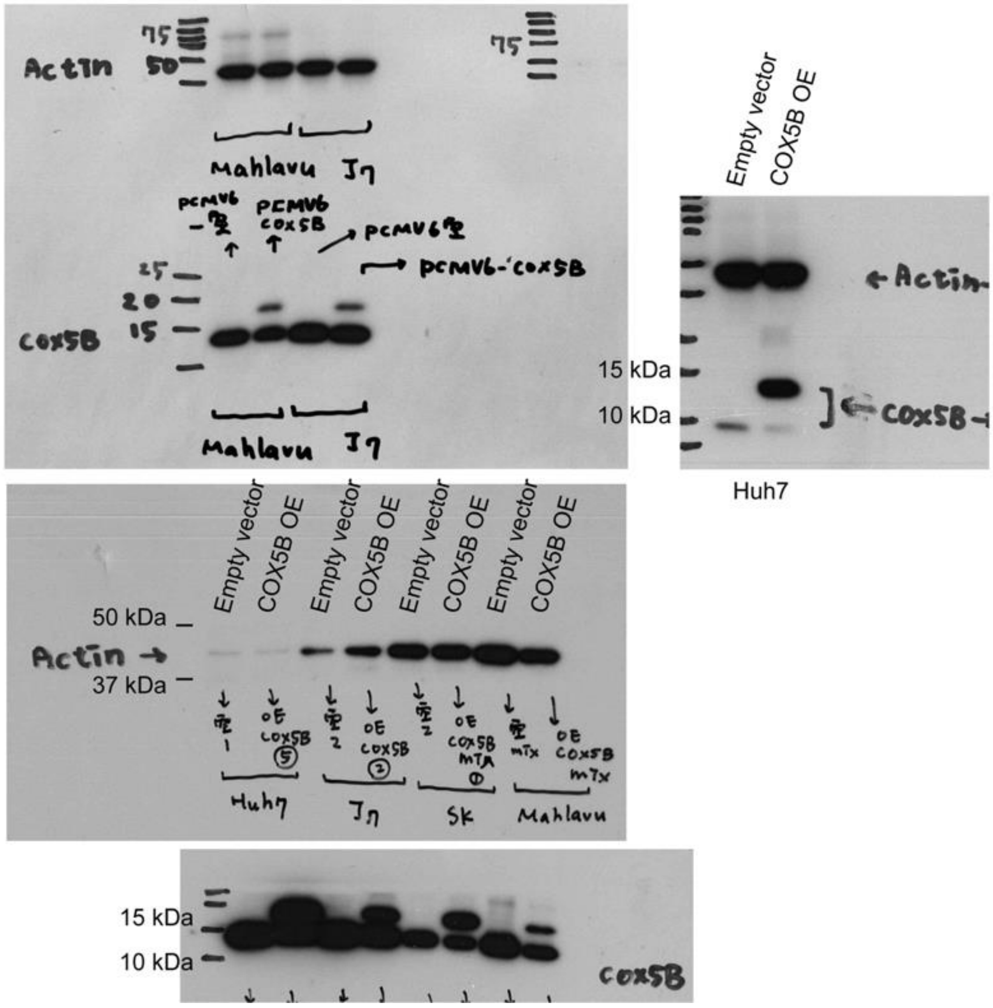

The western blot raw images for Figure 3

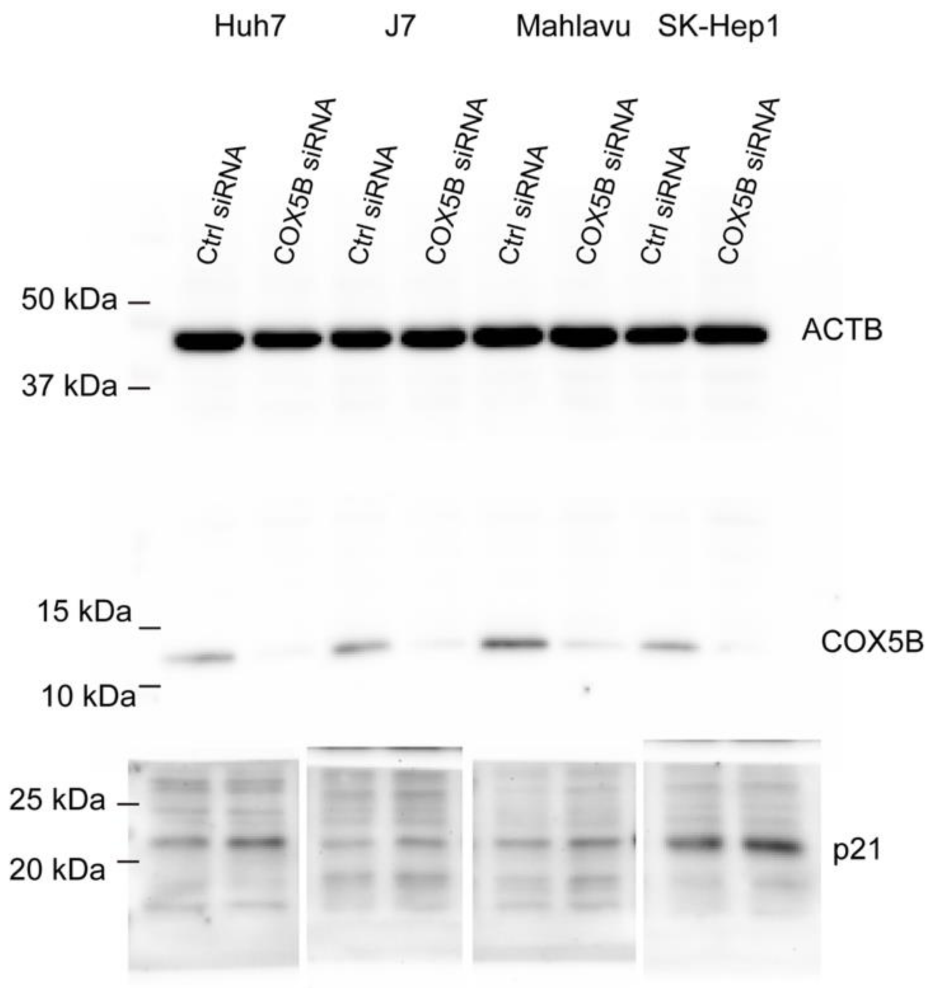
Huh7
J7
Mahlavu
SK-Hep1
Ctrl siRNA
COX5B siRNA
Ctrl siRNA
COX5B siRNA
Ctrl siRNA
COX5B siRNA
Ctrl siRNA
COX5B siRNA
50 kDa
37 kDa
ACTB
15 kDa
10 kDa
COX5B
25 kDa
20 kDa
p21

The western blot raw images for Figure 4B

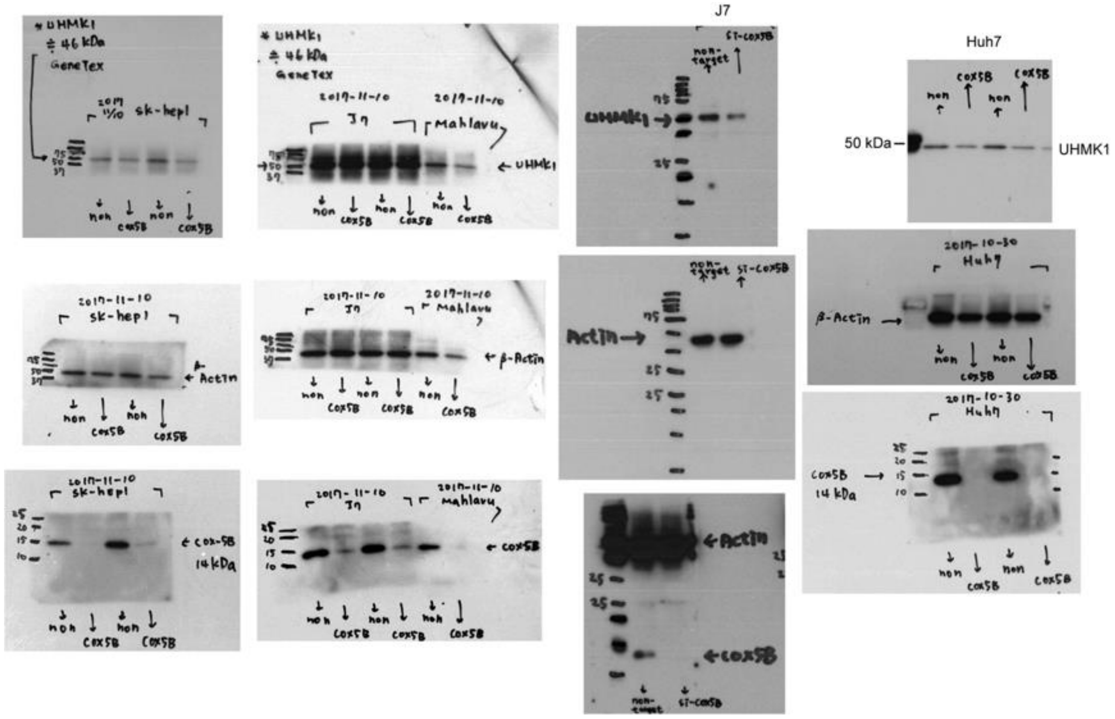

The western blot raw images for Figure 4D

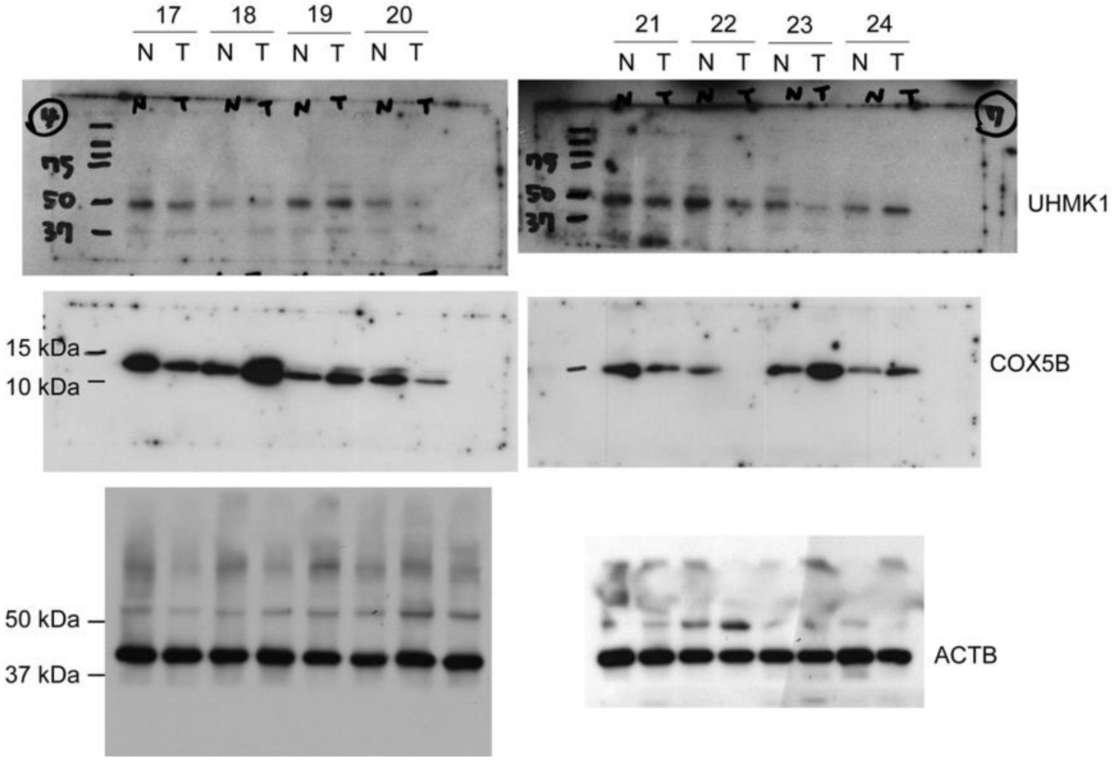

The western blot raw images for Figure 6A-C

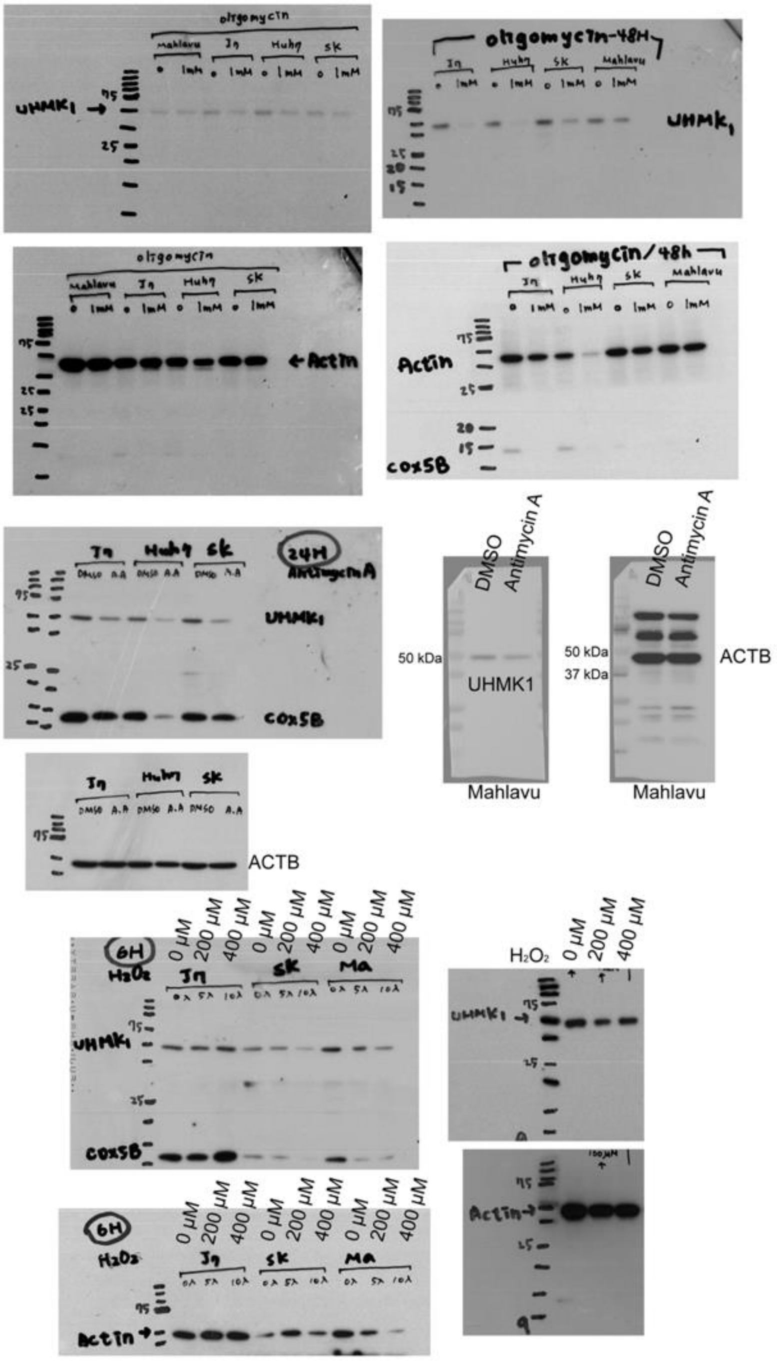

The western blot raw images for Figure 6D

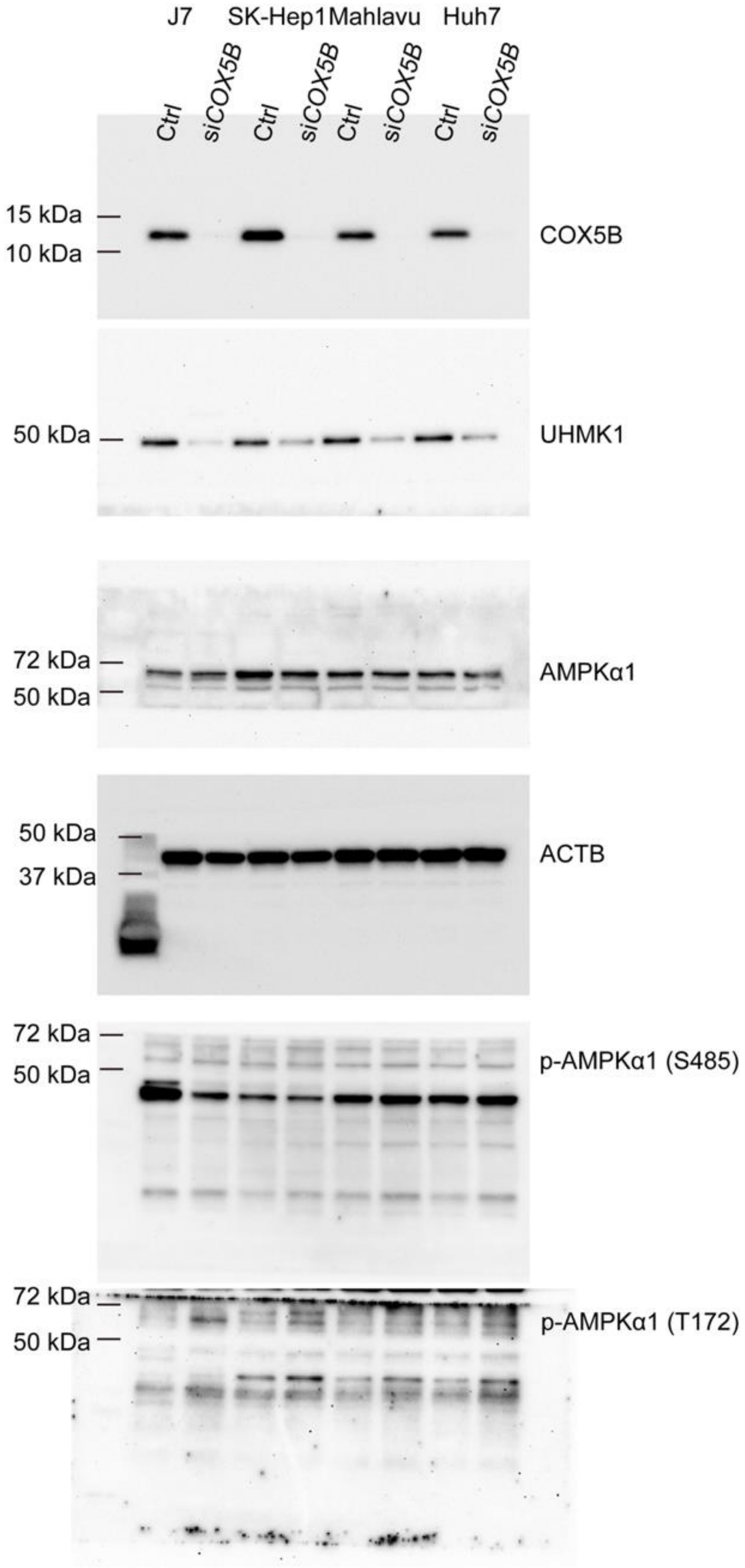

The western blot raw images for Figure 6E

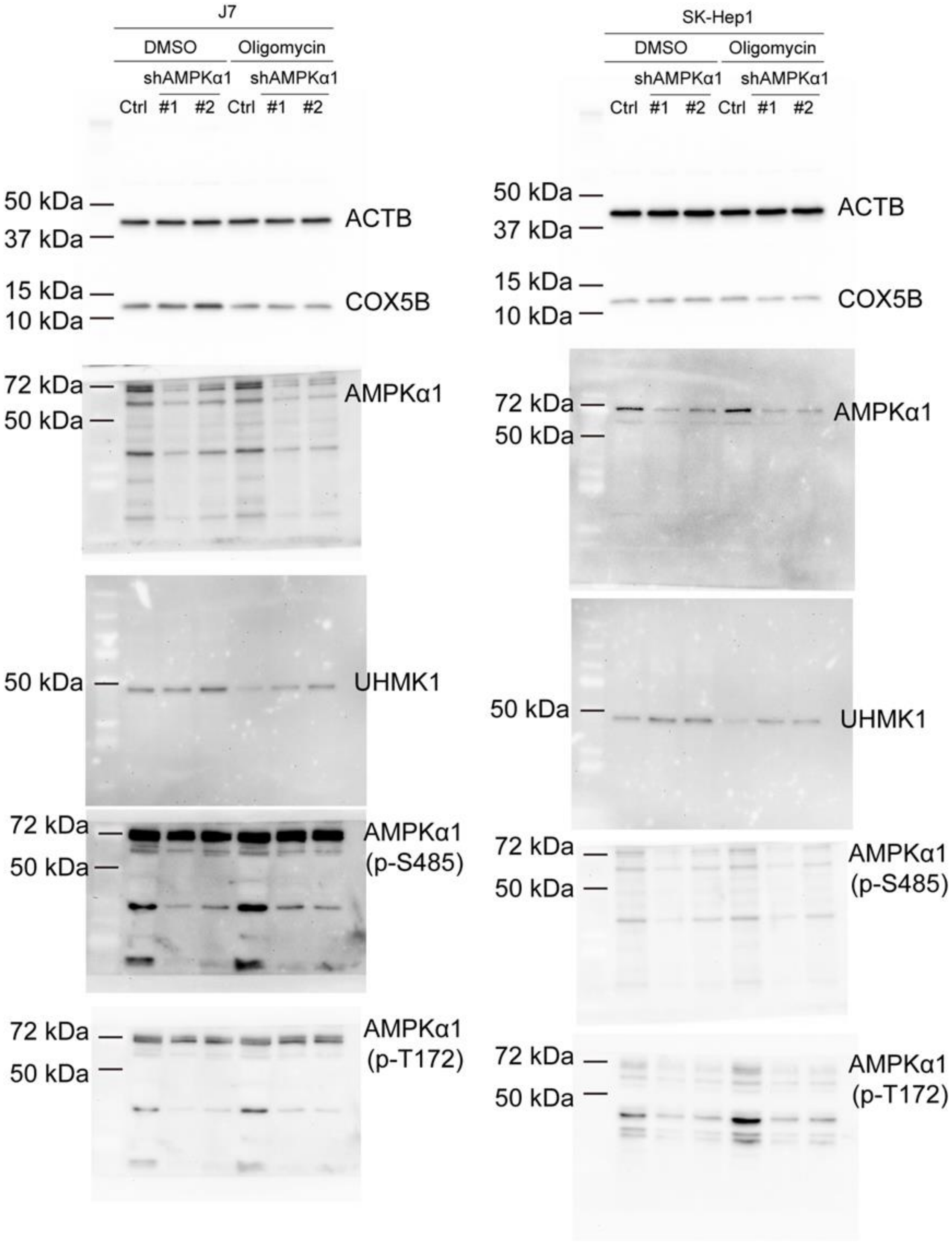

The western blot raw images for Figure 6F

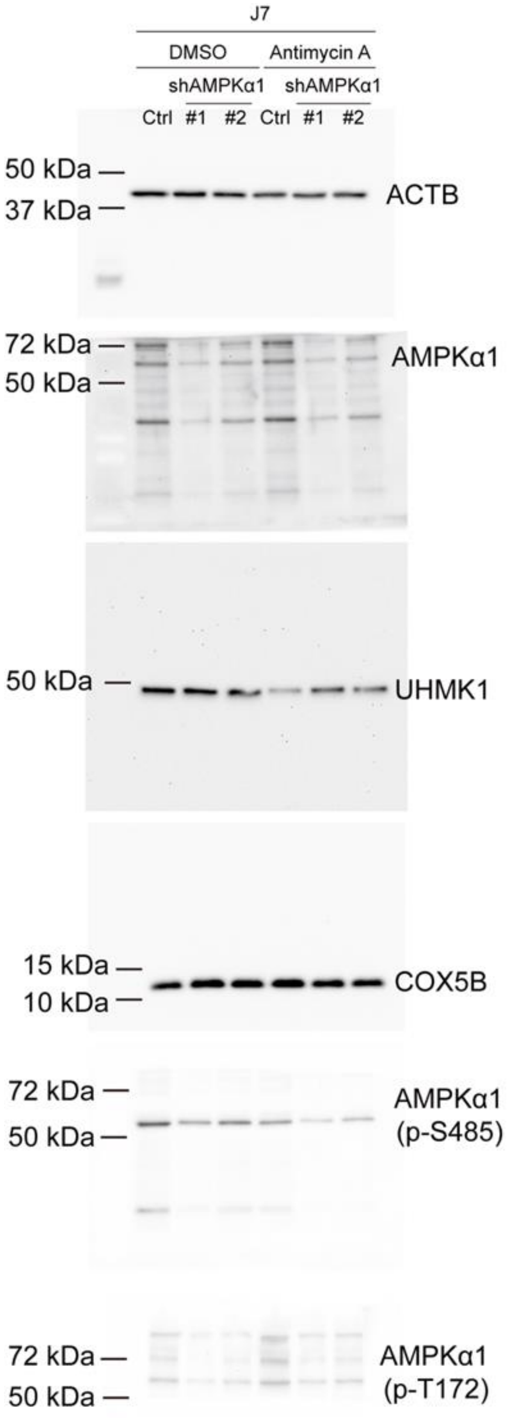

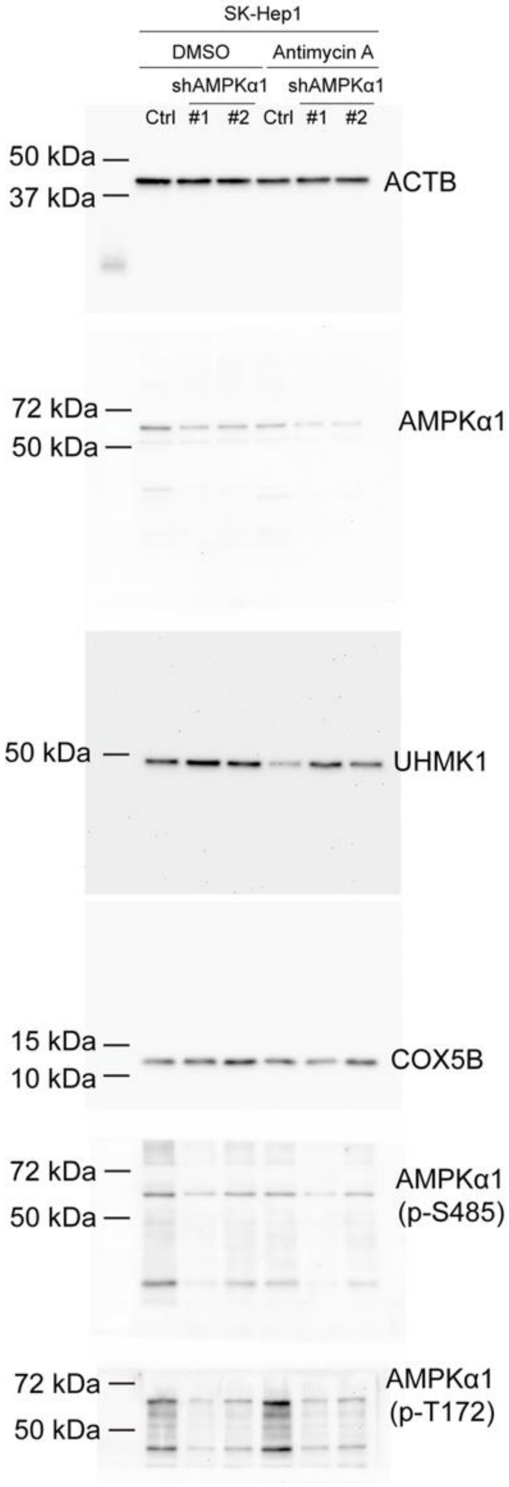

The western blot raw images for Figure 7A

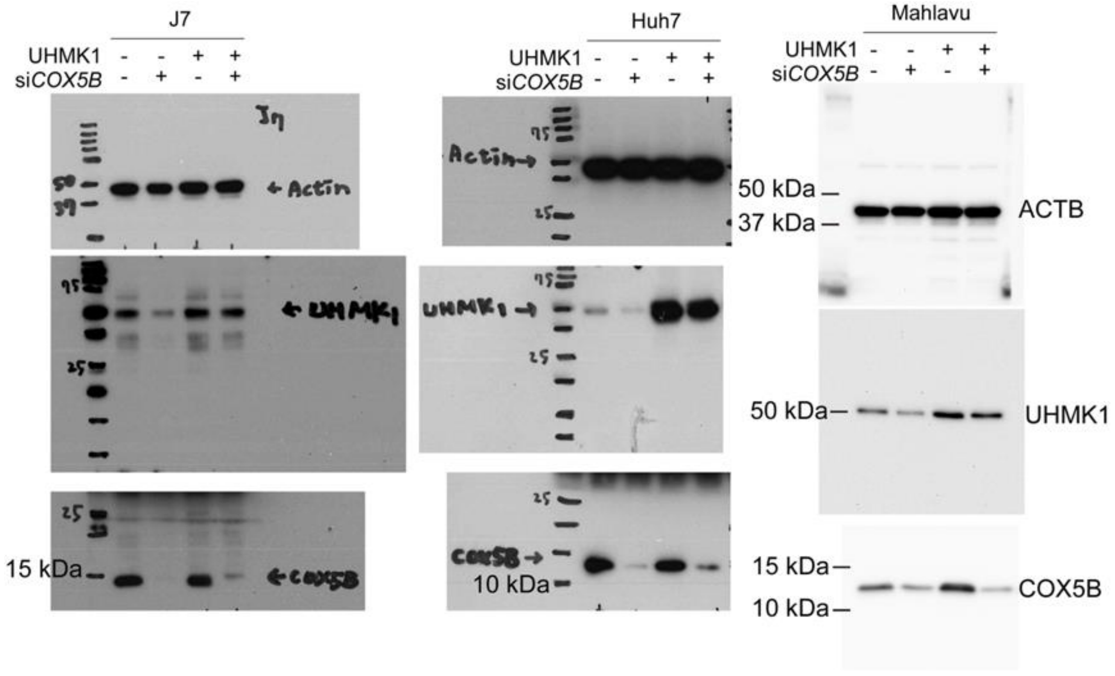

The western blot raw images for Figure 7E

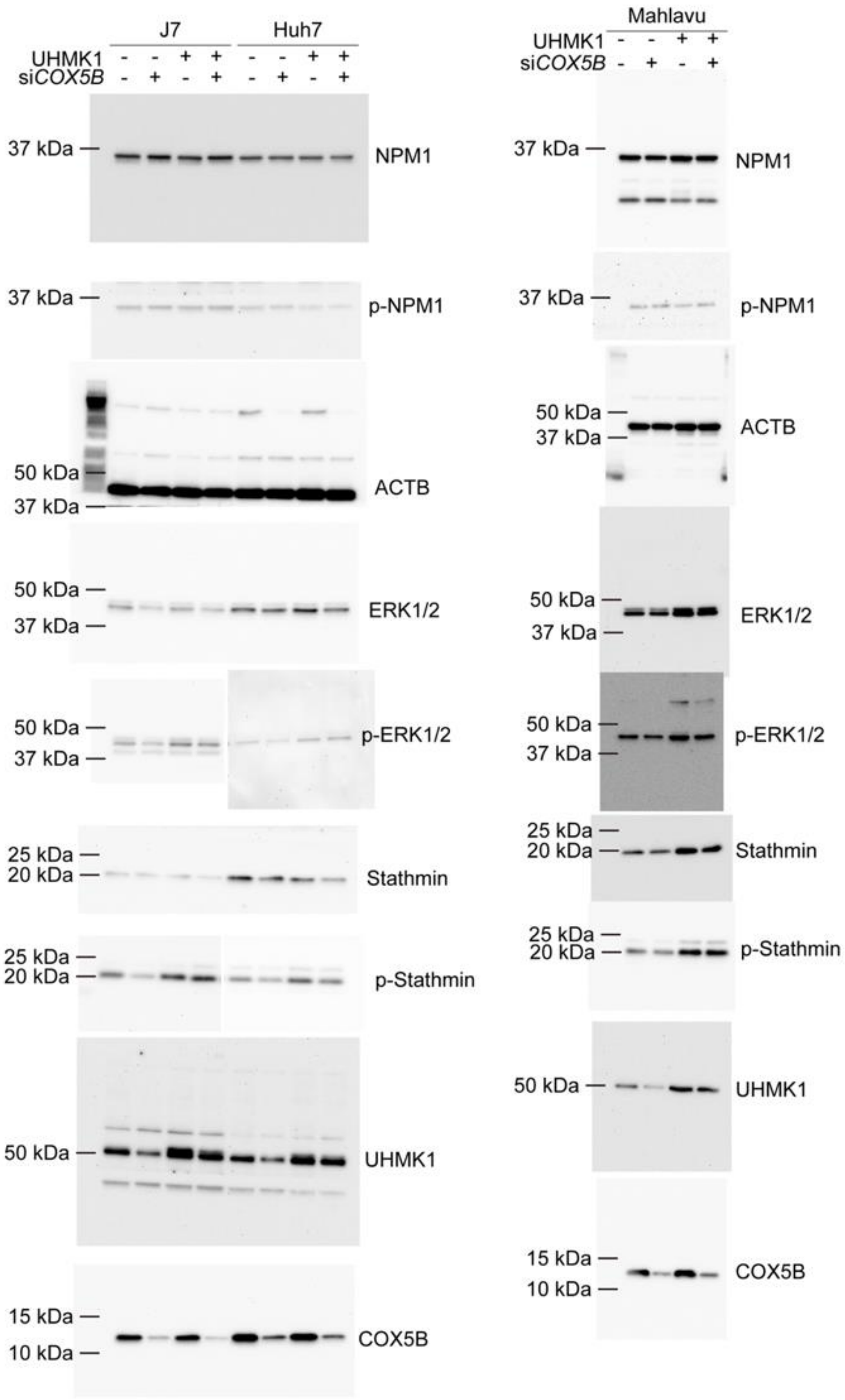

The western blot raw images for Figure 8A

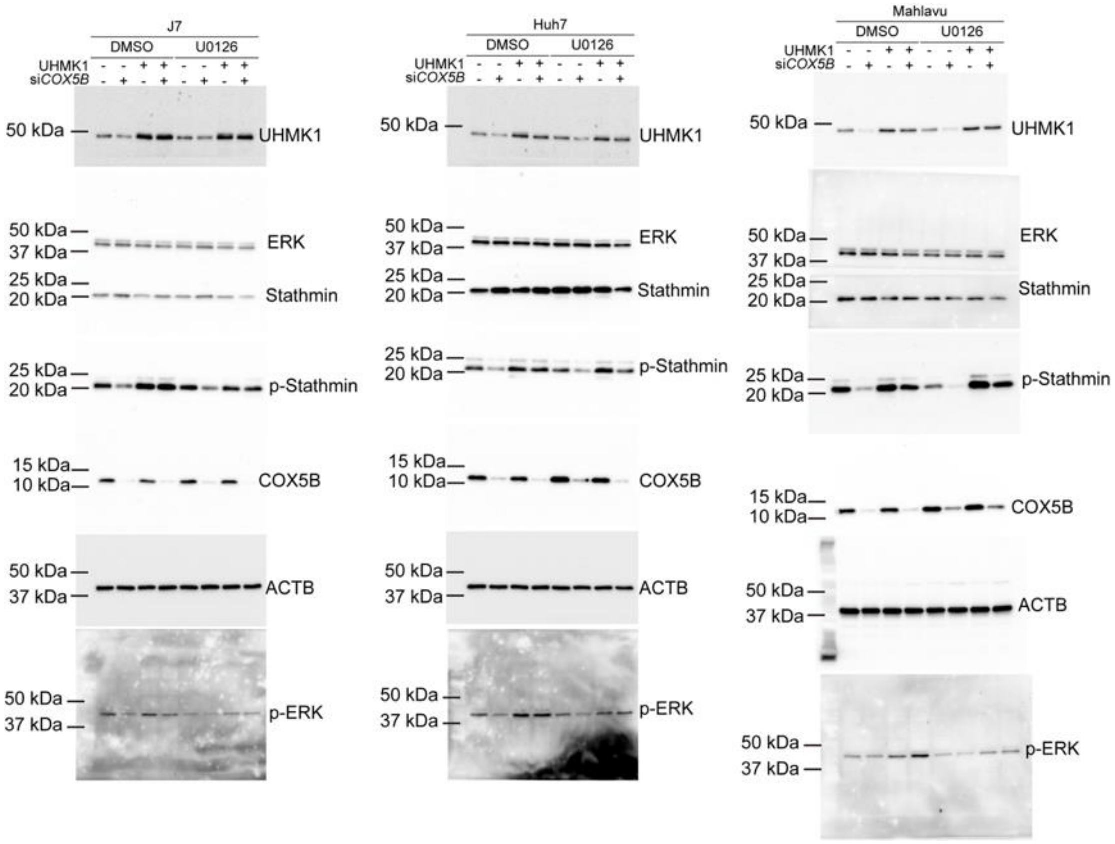

Supplement: Supplementary file 1 [file cancers-12-01646-s001.pdf]
